# Early diagnosis of Alzheimer’s disease using combined features from voxel-based morphometry and cortical, subcortical, and hippocampus regions of MRI T1 brain images

**DOI:** 10.1371/journal.pone.0222446

**Published:** 2019-10-04

**Authors:** Yubraj Gupta, Kun Ho Lee, Kyu Yeong Choi, Jang Jae Lee, Byeong Chae Kim, Goo Rak Kwon, the National Research Center for Dementia, Alzheimer’s Disease Neuroimaging Initiative

**Affiliations:** 1 School of Information Communication Engineering, Chosun University, Gwangju, Republic of Korea; 2 National Research Center for Dementia, Chosun University, Gwangju, Republic of Korea; 3 Department of Biomedical Science, College of Natural Sciences, Chosun University, Gwangju, Republic of Korea; 4 Department of Neurology, Chonnam National University Medical School, Gwangju, Republic of Korea; Arizona State University & Santa Fe Institute, UNITED STATES

## Abstract

In recent years, several high-dimensional, accurate, and effective classification methods have been proposed for the automatic discrimination of the subject between Alzheimer’s disease (AD) or its prodromal phase {i.e., mild cognitive impairment (MCI)} and healthy control (HC) persons based on T1-weighted structural magnetic resonance imaging (sMRI). These methods emphasis only on using the individual feature from sMRI images for the classification of AD, MCI, and HC subjects and their achieved classification accuracy is low. However, latest multimodal studies have shown that combining multiple features from different sMRI analysis techniques can improve the classification accuracy for these types of subjects. In this paper, we propose a novel classification technique that precisely distinguishes individuals with AD, aAD (stable MCI, who had not converted to AD within a 36-month time period), and mAD (MCI caused by AD, who had converted to AD within a 36-month time period) from HC individuals. The proposed method combines three different features extracted from structural MR (sMR) images using voxel-based morphometry (VBM), hippocampal volume (HV), and cortical and subcortical segmented region techniques. Three classification experiments were performed (AD vs. HC, aAD vs. mAD, and HC vs. mAD) with 326 subjects (171 elderly controls and 81 AD, 35 aAD, and 39 mAD patients). For the development and validation of the proposed classification method, we acquired the sMR images from the dataset of the National Research Center for Dementia (NRCD). A five-fold cross-validation technique was applied to find the optimal hyperparameters for the classifier, and the classification performance was compared by using three well-known classifiers: K-nearest neighbor, support vector machine, and random forest. Overall, the proposed model with the SVM classifier achieved the best performance on the NRCD dataset. For the individual feature, the VBM technique provided the best results followed by the HV technique. However, the use of combined features improved the classification accuracy and predictive power for the early classification of AD compared to the use of individual features. The most stable and reliable classification results were achieved when combining all extracted features. Additionally, to analyze the efficiency of the proposed model, we used the Alzheimer’s Disease Neuroimaging Initiative (ADNI) dataset to compare the classification performance of the proposed model with those of several state-of-the-art methods.

## Introduction

Alzheimer’s disease (AD) is a growing or progressive neurodegenerative brain disorder disease. AD is considered to be the most common type of dementia (mental illness), accounting for 50%–80% of dementia cases. It is a complex disease categorized by the accumulation of β-amyloid A(β) plaques and neurofibrillary tangles [1], which are composed of tau amyloid fibrils linked to synapse dysfunction loss and a progressive neurodegeneration, leading to memory loss and other brain-related cognitive problems. The pathophysiological changes that cause cognitive, functional and behavioral impairment in AD patients are thought to begin several years or even decades prior to the beginning of clinical symptoms. AD is typically diagnosed in people above 65 years of age, and it has been stated that the number of the AD patients tends to double every 5 years after the patient age is over 65 years [2]. It is believed that one in every 85 peoples will be affected by the AD disease by the year 2050 [3]. The average life expectancy of AD convalescent patients varies between 3 and 10 years, depending on the age when they were diagnosed with AD. The median lifespan is as long as 7 to 10 years for AD patients whose conditions were identified when they were in their 60s or early 70s. This number decreases to 3 years or less for AD patients who were diagnosed when they were in their 90s [4]. Recently, more specific research criteria have been proposed for the early and accurate diagnosis of AD in the prodromal stage or mild cognitive impairment (MCI) of the disease [5], which is of great importance for the timely treatment and possible delay of the disease.

MCI is often used to refer to patients with objective cognitive impairment who have normal capabilities for the activities of daily living and do not meet the criteria for cognitive decline or dementia [6,7]. Generally, two types of clinical changes are observed in MCI patients over time. First, some MCI subjects will develop AD eventually (i.e., MCI caused by AD (mAD) or MCI converters (MCIc)), whereas others will never develop AD (i.e., stable MCI (aAD) or MCI non-converters). About 35% of MCI patients progress to AD or dementia within a 3-year follow-up period, with a yearly conversion rate of 5%–10%. Predictors of this conversion include whether the patients are carriers of ε4 alleles of the apolipoprotein E (APOE) gene, brain atrophy, clinical severity, patterns of cerebrospinal fluid (CSF) biomarkers, cerebral glucose metabolism, and A(β) deposition [8,9]. aAD and mAD subjects are distinguished by the severity of amnestic impairment with aAD requiring memory loss test performance greater than 1.5 SD (or greater below the age-adjusted mean) below standardized norms on memory tests and mAD requiring memory loss test performance between 1.0 and 1.5 SD below standardized norms [10,11]. aAD patients have milder verbal memory impairment than mAD and are thought to represent a very early stage of the disease that may be optimal for disease modification treatments. These subtypes of MCI difference may have implications for differences in biomarker abnormalities, likely clinical course, and treatment response in patients with cognitive impairment [10]. Because of the heterogeneity of the clinical presentation and underlying etiologies within the MCI group, there is no documented treatment for MCI subjects. Furthermore, the clinical course of MCI is more heterogeneous than distinctive. However, there are many causes of MCI and not all are related to progressive neurodegenerative disorders. Therefore, diagnosing the underlying etiology is very challenging for individuals with cognitive impairment and there is a need for more accurate diagnostic tests to identify MCI patients in whom AD may be the underlying cause. As early as possible this diagnosis methods should be performed in the course of the disease [12]. This problem is a qualitative prognosis problem that can be solved via the classification between aAD and mAD. Because AD is a progressive neurodegenerative disease, there are continuous changes between previously measured and current clinical scores (e.g., AD Assessment Scale Cognitive Subscale (ADAS-Cog) and Mini-Mental State Examination (MMSE)). Therefore, it is important to predict future clinical scores based on data from earlier time points, which is particularly helpful for monitoring disease progression. However, the classification between aAD and mAD is challenging and benchmark results in [12,13] showed low accuracy which, according to the author, was likely caused by the heterogeneity of cortical thinning patterns in aAD subjects. We can overcome this limitation by increasing the number of training samples to cover all complex patterns or by choosing only desirable features to reflect the differences between these two groups.

Over the past few years, many high-dimensional pattern-based classification methods have been developed for AD and MCI. These methods have largely focused on the individual modalities of biomarkers for the diagnosis of AD or MCI (e.g., structural brain atrophy can be measured by structural magnetic resonance imaging (sMRI) [13–17], metabolic brain alterations can be measured by fluorodeoxyglucose positron emission tomography (FDG-PET) imaging [18,19], and pathological amyloid depositions can be measured from CSF [20,21]), which may affect the overall classification performance because different modalities of biomarkers provide different complementary information, which are useful for the diagnosis of AD [20–23]. All the above criteria are based on clinical scores of early episodic memory impairment with the presence of at least one extra supportive feature, including abnormal sMRI results or abnormal CSF amyloid and tau biomarkers. Previous experiments have shown that the use of multiple biomarkers yields promising results for the early diagnosis of AD or MCI. Moreover, these studies have combined sMRI-based markers with biomarkers based on FDG-PET [22,24], CSF [21,23,25], APOE [26] genotype, or combinations of these biomarkers [27,28] to achieve promising results. However, the availability of all four biomarkers (CSF, PET, sMRI, and APOE genotype) is limited in clinical practice because obtaining these measurements is laborious for both patients and doctors. Moreover, measurements obtained from CSF and FDG-PET are considered an invasive [29,30]. Recent studies focusing only on sMRI modality have achieved a high classification accuracy of 80%-99% in identifying healthy controls (HC) from AD subjects [14–16,29,31–33] and 70%-90% when predicting aAD vs. mAD [13,29]. Methods in which features are extracted from sMR images for existing classification approaches can be divided into three groups: voxel-based morphometry [34–36], cortical thickness [27–30,35–39], and region of interest (ROI) (hippocampal volume) [40–43] methods. It has been shown that the most effective features for AD or MCI classification are extracted from ROIs around the hippocampus, entorhinal cortex, parahippocampal gyrus, amygdala, etc. [13,44–46]. This type of ROI features can improve the classification accuracy, and it also helps to reduce the number of false positive diagnoses.

In this study, we used images from the National Research Center for Dementia (NRCD) dataset to achieve the goals of our proposed method, and also to classify AD with other groups. Here, three different feature extracted from sMRI images were combined into one as stated above, namely, voxel-based morphometry (VBM), hippocampal volume (HV), and cortical and subcortical segmented region (volume, thickness, meancurve, Foldcurv, Curvind, and Gauscurv) (CSC) methods, for the early classification of AD or MCI. This paper shows the benefit of combining all three features into one for the early diagnosis of AD or MCI patients. We used all 326 subjects and their corresponding T1-weighted 3-T MR images from the NRCD dataset to differentiate AD, MCI, and HC individuals. To analyze the impact of combined features compared to that of individual features on the given classification problems, we used three different types of classifier, i.e., K-nearest neighbor (KNN), random forest (RF), and support vector machine (SVM), to evaluate the area under the curve of the receiver operating characteristic curve (AUC-ROC), classification accuracy (ACC), sensitivity (SEN), specificity (SPE), precision (PRE), and F1-score (F1) in each experiment. We also measured the Cohen’s kappa statistic and McNemar’s chi-squared test value for each classification group. These were the statistical analysis tools. The obtained experimental result showed that the use of combined features from the VBM, HV and CSC techniques yielded superior performance in terms of AD or MCI classification compared to the use of individual features.

## Materials and methods

### Ethics statement

In this study, all procedures performed involving human contributors were in accordance with the latest Declaration of Helsinki. Subjects were prospectively recruited from two centers: Chonnam National University Hospital, and Chosun University Hospital including the National Research Center for Dementia in Gwangju, Korea. All patients provided written informed consent at the time of inclusion in the cohort for use of data, samples, and images before the data collection. In the case of AD patients with the inability of consent, the family member of subjects gave consent before the participation. Assessments or psychological tests were not used to determine whether a patient were able to provide written informed consent. The consent procedure and data acquisition were approved by the Institutional Review Board (IRB number 2013-12-018-070) of Chonnam National University Hospital, and Chosun University Hospital, Gwangju, South Korea.

### Subjects

In this study, the dataset was acquired from a pool of persons registered at the National Research Center for Dementia in Gwangju, Korea, from January 2014 to March 2018. All subjects were examined by skilled neurologists and received a full dementia screening test, which included past history, neurological examination, laboratory and neuropsychological tests, and brain MRI. All subjects were examined through clinical interview, which included an assessment of the Clinical Dementia Rating (CDR) [47]. All HC subjects received a CDR score of 0. This group had a normal range of cognitive function and good general health with no sign of brain atrophy changes on sMRI scans, which were analyzed with the Analyze 11.0 software on an iMac system running OS X Server. All aAD (stable MCI that did not convert to AD within a 36-month time period) subjects received a CDR score of 1, and their neuropsychological assessment z-scores were above -1.5 according to education-, age-, and gender-specific norms. They had good health overall with no widespread multiple lacunae or other focal brain lesions or brain atrophy other than supposed incipient AD on MRI scans. All the mAD (MCI that converted to clinical AD within a 36-month time period) subjects met [47] and received a CDR score of 2. Their neuropsychological assessment z-scores were below -1.5 on at least one of the memory assessments according to the education-, age-, and gender-specific norms. We did not consider MCI patients who had been followed for less than 36 months and had not converted within this time frame. All the AD subjects received a CDR score of 3. This group had an affected cognitive function, had severe memory loss, and also showed evidence of brain atrophy changes in sMRI scans. The diagnosis of AD, aAD, mAD, and HC patients was made according to the clinical criteria proposed by the NIA-AA or IWG-2 on AD, MCI and HC [5–7,9]. The patients were also unable to make judgments or solve given problems. The exclusion criteria were (1) serve vision or hearing loss; (2) illiteracy; (3) sign of focal brain lesions on sMRI including multiple lacunae and WM hyperintensity lesions of grade 2 or more according to the Fazeka scale; (4) any other type of dementia; (5) any significant medical, neurological, or psychiatric disorders that could disturb cognitive function; and (6) present use of psychoactive medication. More detailed information about the participants had been reported before in [48,49]. The subjects were between 49 and 87 years of age, spoke Korean, and had been studied sufficiently to give a reasonable evaluation of functionality. As [5,6,50,51] suggest that for aAD subject group the objective memory loss measured by education score-adjusted on Wechsler Memory Scale Logical Memory II subscale: 9–11 for 16 or more years of education, 5–9 for 8–15 years of education, and 3–6 for 0–7 years of education. Whereas for the mAD subject group the objective memory loss measured by education score-adjusted on Wechsler Memory Scale Logical Memory II subscale: ≤ 8 for 16 or more years of education, ≤ 4 for 8–15 years of education, and ≤ 2 for 0–7 years of education. Therefore, we have followed above-mentioned criteria to give an educational score to the patients. Here, means and standard deviations were calculated to describe continuous variables. Table 1 shows the demographic information for the 326 subjects. We selected all the patients for whom processed images were available. A total of 326 subjects were selected with 81 subjects belonging to the AD group (42 females, 39 males; age ± SD = 71.86 ± 7.09 years, education level = 7.34 ± 4.88 years, range = 56–83 years; range = 0–18 years), 171 subjects belonging to the HC group (88 females, 83 males; age ± SD = 71.66 ± 5.43 years, range = 60–85 years; education level = 9.16 ± 5.54 years, range = 0–22 years), 39 subjects belonging to the mAD group (14 females, 25 males; age ± SD = 73.23 ± 7.09 years, range = 49–87 years; education level = 8.20 ± 5.19 years, range = 0–18 years), and 35 subjects belonging to the aAD group (20 females, 15 males; age ± SD = 72.74 ± 4.82 years, range = 61–83 years; education level = 7.88 ± 6.30 years, range = 0–18 years).

**Table 1:** Subject demographic information (from the NRCD dataset).

Diagnostics	Number of Subjects	Age Mean ± SD (years)	Gender		Education Mean ± SD (years)
			F	M	
**AD**	81	71.96* 7.08 [56–83]	42	39	7.34* 4.86 [0–18]
**aAD**	35	72.72* 4.82 [61–83]	20	15	7.88* 6.30 0–18]
**mAD**	39	73.24* 7.44 [49–87]	14	25	8.20* 5.19 0–18]
**HC**	171	71.66* 5.43 [60–85]	88	83	9.16* 5.54 0–18]

*Values are shown as a mean ± standard deviation [range].

From Table 1 we can see that aAD (stable MCI) patients ages were between 61–83 and their education score was ranged from 7.88* 6.30 0–18], likewise, mAD (who had converted to AD within a 36-month time period) patients ages were between 49–87 and their education score was ranged from 8.20* 5.19 0–18], which show that mAD subject groups were more educated then aAD subject groups and mAD subjects were young compared to aAD subject groups. Student’s unpaired *t*-tests were applied to decide whether there were statistically significant differences between the demographics and the clinical characteristics, and a standard *p-*value (0.05) was used as a significance threshold level. No significant differences were found in any group. Except for the mAD group, all groups included a large number of female subjects. Compared to the other groups, the mAD group had older subjects. As can be seen from Table 1, the AD group had a much lower level of education compared to that of other groups. To obtain unbiased estimations of the performance, we randomly split the dataset into two groups: a training dataset and a testing dataset, at a ratio of 70:30, respectively. The classifiers were then trained on the training dataset, and the measurements of the diagnostic AUC, ACC, SEN, SPE, PRE, F1 and Cohen’s kappa were calculated independently using the testing data.

### sMR image acquisition

The images used in this study were T1-weighted sMR images, which were acquired using a 3D magnetization-prepared rapid the gradient-echo sequence with a resolution of (1 × 1 × 1) millimeter voxel size. The 3-T T1-weighted axial images were obtained with the following parameters: slice thickness, 5.0 mm; interslice thickness, 1 mm; repetition time, 2000 ms; echo time, 20 ms; flip angle, 90°; matrix size, 324×244 pixels; and field of view, 183×220 mm^2^.

### Image analysis

**Fig 1:**
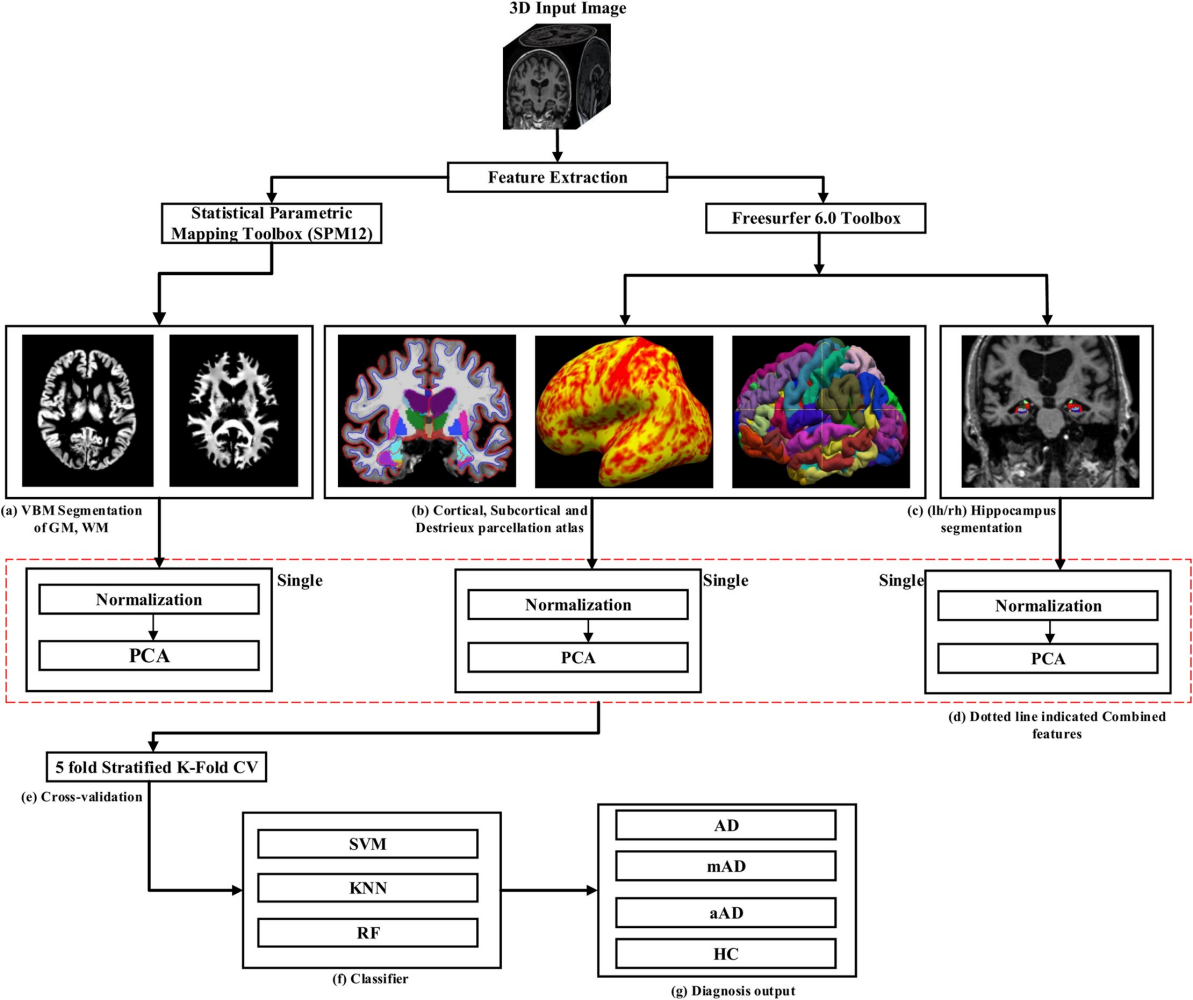
Block diagram of the proposed method. (a) VBM segmentation of GM, WM, and CSF; (b) cortical, subcortical, and Destrieux parcellation atlas; (c) (lh) and (rh) hippocampus segmentation; (d) combined features; (e) cross validation (CV); (f) classifier; and (g) diagnosis output.

An image preprocessing step was applied to each sMR image. It is well known that strong bias fields can cause serious mislabeling of voxel tissue types, which can compromise the accuracy of the techniques that rely heavily on tissue density (e.g., registration) and, in particular, on gray and white matter contrasts. Therefore, to minimize this effect, we applied an N4 bias field correction method using the Advanced Normalization Tools (ANTs) [52] toolbox to correct the inhomogeneity artifacts in each image. Then, feature extraction process and a feature selection (selection of high-level features) process were applied. These features contain the most essential information for the early classification of AD or MCI. In this study, a 3D sMR input image was fed into a feature extraction toolbox to extract the clinical features (volume, thickness, etc.). Fig 1 shows a systematic block diagram for the proposed method. In this experiment, we used two different types of toolboxes for the extraction of features: Statistical Parametric Mapping (SPM version 12) and Freesurfer (version 6.0). Lastly, the extracted features were passed to the classifier to measure the classification performance between different groups.

### Feature extraction

It is well known that ROI [45] features are the most effective features for the classification of AD or MCI. In the proposed technique, we used SPM12 and Freesurfer to extract three well-known features (VBM, CSC, and HV) from each sMR image. Both these tools are fully automated in nature. They were applied on the 3-T T1-weighted sMR images, which were acquired from the NRCD dataset.

#### Voxel-based morphometry

**Fig 2:**
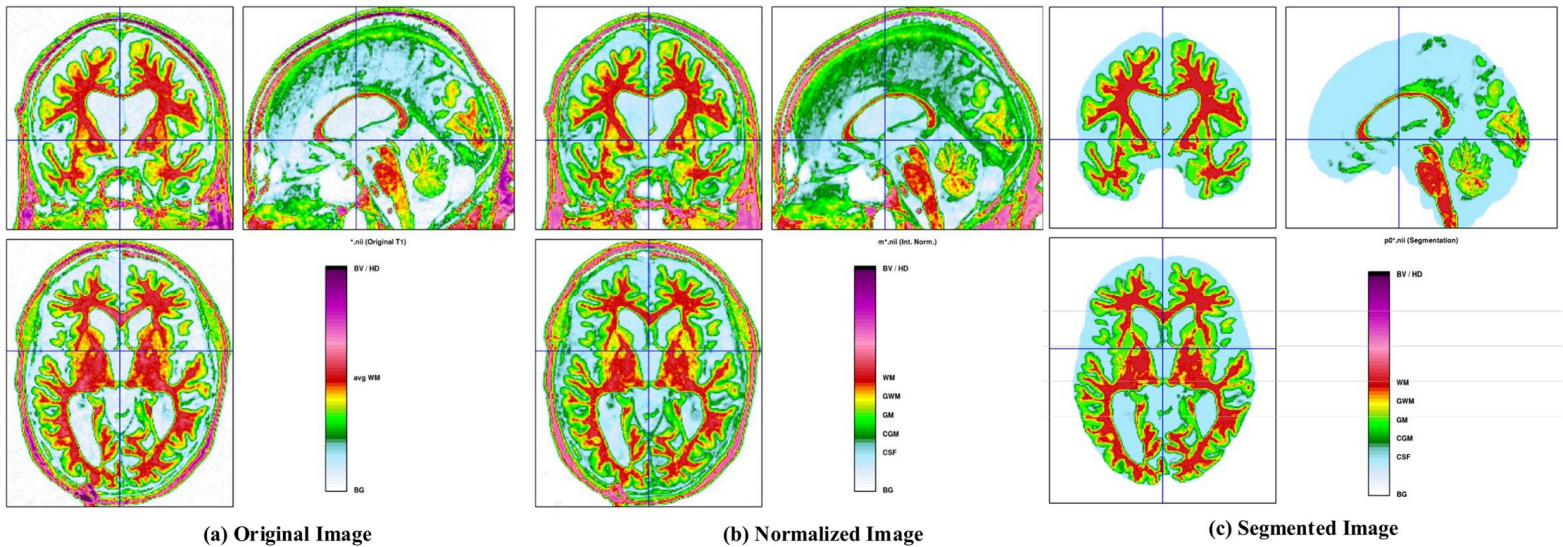
**CAT12 VBM images showing segmented brain tissue:** (a) original image, (b) normalized image with template, and (c) brain tissue segmented image.

VBM is a neuroimaging analysis method that allows researchers to investigate focal differences in brain anatomy using statistical tools. Morphometry analysis has become an important tool for performing quantitative measurements and identifying structural differences throughout the brain. The significance of the VBM method is that it is not partial to particular subjects and provides an unbiased and comprehensive score that represent the anatomical differences between groups throughout the brain [53]. The MRI data for VBM are provided as 3D volumetric T1-weighted images. VBM essentially uses statistical tests on all voxels in the brain images to identify the volume differences between groups. For example, to recognize the differences in the patterns of the regional anatomy between two groups of subjects, one can perform a series of *t*-tests on each voxel in each image. Additionally, sMRI measurements of brain atrophy are promising biomarkers for tracking disease progression in AD patients. Recently, VBM has been applied in a number of studies [54–56] for the detection of AD. It can be used to study the volumetric atrophy of the gray matter (GM) that exists in the neocortex of the brain, which can be used to distinguish AD patients from HC individuals. There are many toolboxes available for performing VBM (SPM12, FSL, 3D Slicer, etc.); however, we select SPM12 and its extension called as Computational Anatomy Toolbox (CAT version 12), which provides computational anatomy functions. These tools can be downloaded from https://www.fil.ion.ucl.ac.uk/spm12/ and http://www.neuro.uni-jena.de/cat/, respectively. CAT12 provides diverse morphometric methods, such as VBM, surface-based morphometry, deformation-based morphometry, and ROI- or label-based morphometry. We chose the CAT12 VBM because it uses SPM12 segmentation by default. First, the sMRI data were anatomically standardized using the 12-parameter affine transformation provided by the SPM template to compensate for the differences in brain size. We chose the East Asian brains template and left all other parameters to their default settings. The sMR images were then segmented into GM, white matter (WM), and CSF images by using a unified tissue segmentation technique after image intensity non uniformity correction was performed. The obtained linearly transformed and segmented images were then nonlinearly transformed using diffeomorphic anatomical registration (DARTEL) techniques and modulated to create a modified template for DARTEL based on the MNI152 template [57], followed by smoothing using an 8 mm full breadth at half maximum kernel. The final step consists of voxel-wise statistical tests. To construct a statistical parametric map, we computed contrast values based on a general linear model estimated regression parameters. This technique implements a two-sample *t*-test approach to determine whether there are significant regional density differences between two groups of GM images. We obtained regional information values representing significant density differences between GM images after performing a false discovery rate (FDR) and a family-wise error rate correction. Based on this information, cluster values can be derived to create ROI binary masks, which were later used to acquire GM volumes from GM images for use as morphometric features. Fig 2 shows the segmented brain tissue images generated using the CAT12 VBM methods.

#### Cortical and subcortical volumetric features

The 3D volumes from all 326 subjects [58–60] (freely available online at http://surfer.nmr.mgh.harvard.edu/, which provides measurable gray-level volume data for various brain structures. For preprocessing using Freesurfer, high-quality T1-weighted MRI data, such as Siemens MPRAGE or General Electric spoiled gradient recalled sequences with resolutions of approximately 1 mm^3^, are needed. We ran Freesurfer without user intervention (using the command “recon-all–I file-name.nii–all”) because this is the process mode that would be used in a preprogrammed pipeline for processing patient data. Cortical and subcortical volumetric features were computed using the cross-sectional automated Freesurfer routine with the default parameters. Bilateral ROIs were joined during the process. In the cortical surface stream, Freesurfer constructs a model of the boundary between WM and cortical GM, as well as between pial surfaces. Once these surface values are known, an array of anatomical calculations is enabled, including CTH, folding, curvature, surface area, and surface normal calculations, for each part of the cortex. The cerebral cortex can be alienated into four sections referred to as lobes. Measurements for all four lobes are extracted by Freesurfer. In this study, we adopted the Desikan-Killiany atlas, which parcellates the entire cortex into 68 labeled regions for each hemisphere. To reduce the size of each feature vector, we combined the cortex regions into four lobes (discussed above) and a cingulate cortex according to the specifications on the Freesurfer website^1^. In this automated subcortical segmentation process, each voxel (in the form of normalized brain volume) is assigned one of 40 labels representing 40 subcortical regions (e.g., amygdala, cerebellum, lateral, and thalamus). It can take up to 11–14 h to complete the subcortical volume segmentation for one subject. Table 2 lists the features extracted from sMRI images using the Freesurfer toolbox.

**Table 2:** Overview of features extracted from sMRI images using the Freesurfer toolbox.

sMRI biomarkers	Selected ROI	Toolbox
**Cortical Parcellation**^1^	Frontal lobe	Freesurfer
	Parietal lobe	
	Temporal lobe	
	Occipital lobe	
	Cingulate lobe	
**Subcortical volumetric features**	Amygdala	Freesurfer
	Lateral	
	Choroid-plexus	
	Thalamus	
	Caudate nucleus	
	Hippocampus	
	Putamen	
	Entire brain	
**Hippocampus subfield**	Left hippocampus hemisphere	Freesurfer
	Right hippocampus hemisphere	

^1^https://surfer.nmr.mgh.harvard.edu/fswiki/CorticalParcellation

#### Hippocampus volume

**Fig 3:**
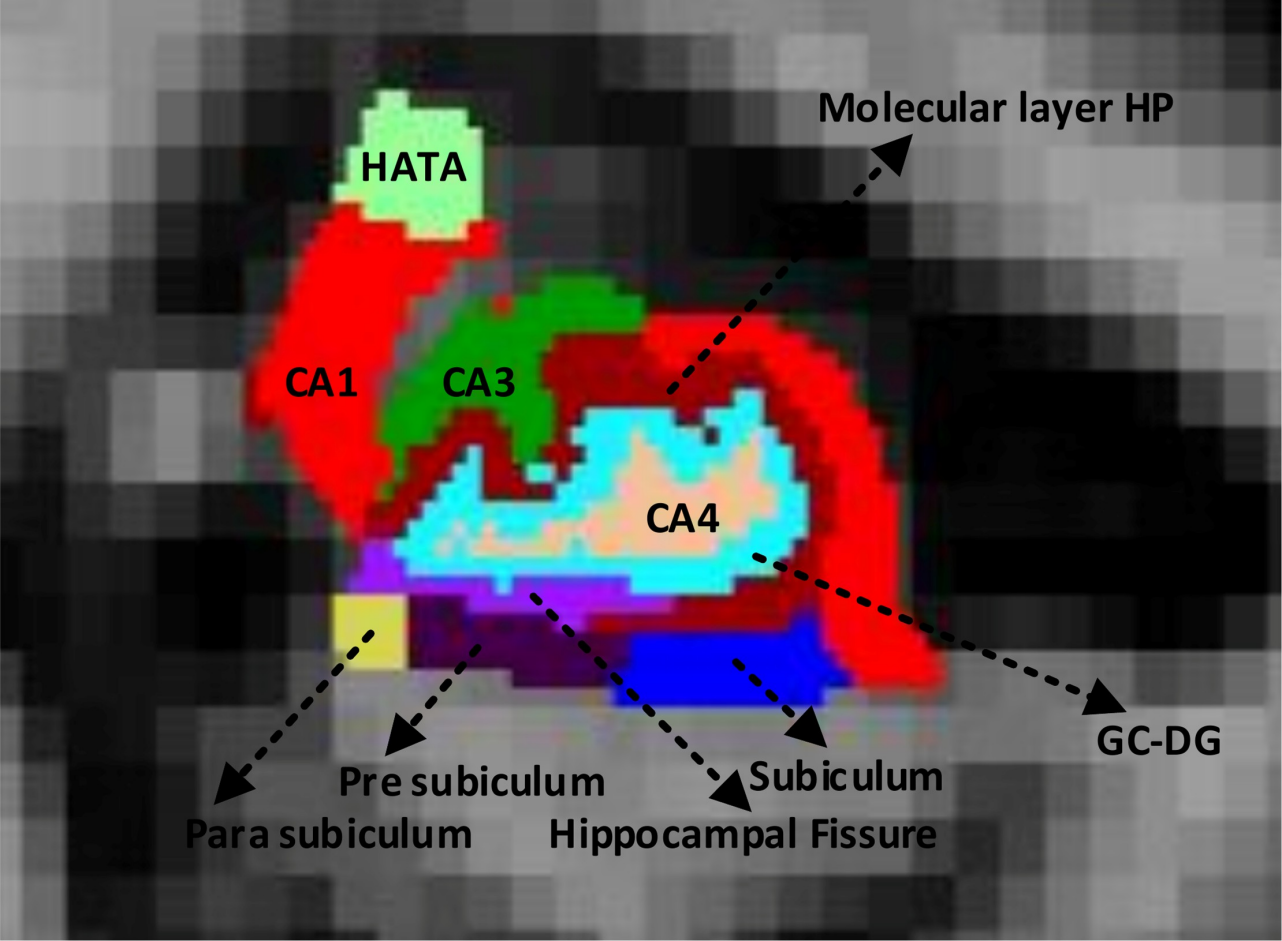
Deep (left and right) segmented regions of the hippocampus from the Freesurfer free-view application.

Segmentation of the HV was performed using Freesurfer [61,62]. This study was inspired by the fact that HV is the most widely used sMRI biomarker for the early detection of AD [63]. Additionally, because Freesurfer segments many ROIs for each subject, it is not restricted to a specific ROI. The left and right hippocampus were both segmented using Freesurfer. This technique estimates the probability that each voxel belongs to a certain structure based on a priori knowledge regarding spatial relationships, which is acquired by using a training set. It uses the differences in voxel intensity to locate and parcellate subcortical structures and to perform affine registration in the Talairach space. The Freesurfer processing stages are detailed in [59], and Fig 3 shows the deep segmented regions of the hippocampus.

In Fig 3, CA1 is the first region along the hippocampal path. A major output path originates from this region and travels to the fifth layer of the entorhinal cortex. From the granule cell of mossy fibers, the CA3 region receives inputs in the dentate gyrus. It also receives inputs from cell in the entorhinal cortex by following the perforant path. The CA3b region [64] occupies the central region between the fimbria and the fornix connection. The CA3c region [64] is located near the dentate and eventually inserts itself into the hilus. Much of the synchronous fragment activity that is associated with interictal epileptiform movement appears to be generated in the CA3 region. The CA4 region is often referred to as a hilar region when considering portions of the dentate gyrus. Unlike the pyramidal neurons in the CA1 and CA3 regions, the neurons in the CA4 region contain mossy cells that receive inputs primarily for the dentate gyrus from granule cells in the form of mossy fibers [61].

## Feature selection

**Fig 4:**
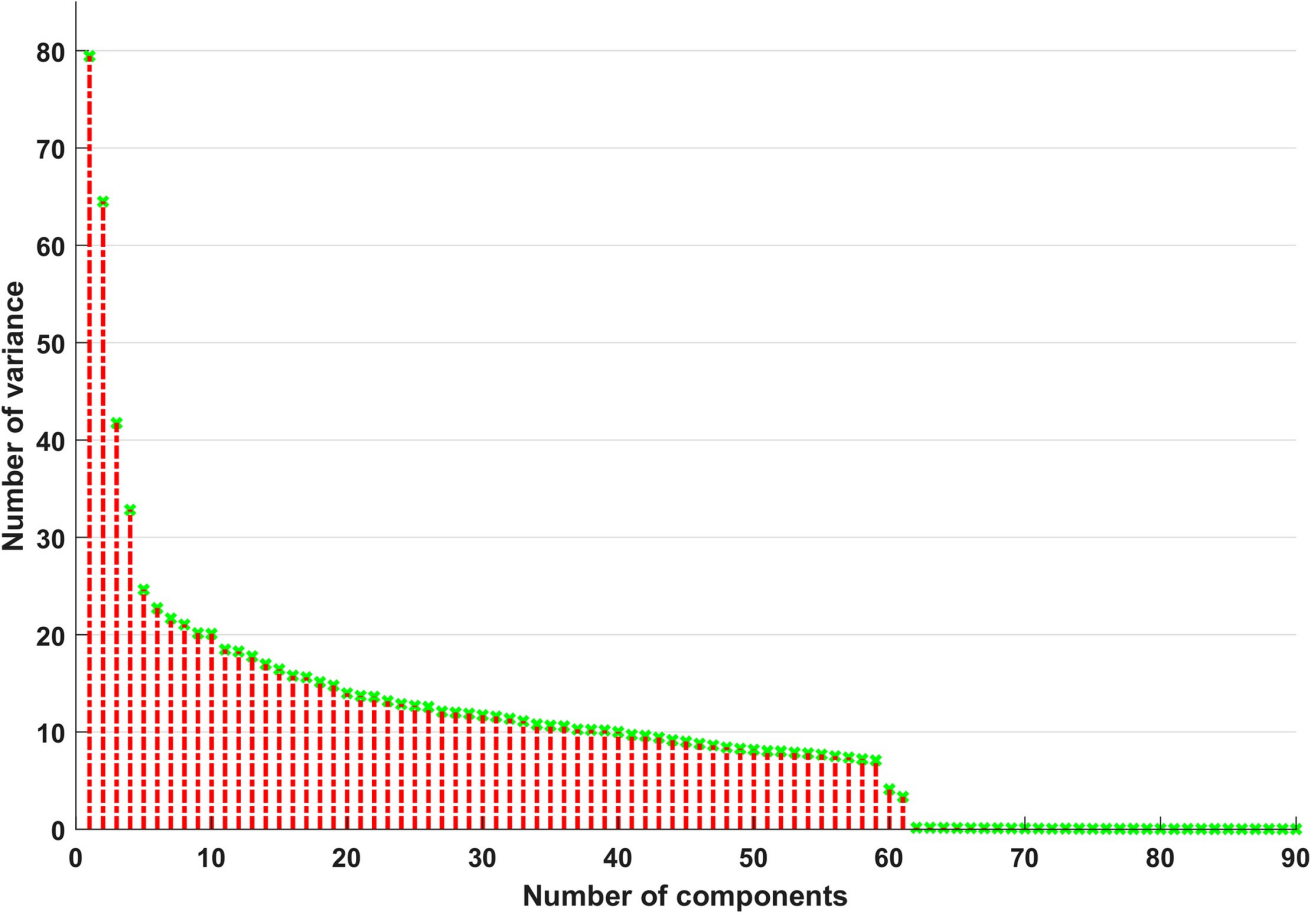
Number of principal components (PCs) vs. No. of variance for AD vs. HC group comparison.

After the feature extraction stage, a normalization process is performed, where all features are normalized to zero mean and unit variance to reduce data redundancy and improve data integrity between features, as shown in Fig 1. Specifically, given a data matrix *X*, where the rows represent the subjects and the columns represent the features, the normalized matrix *X*_*norm*_ with a elements *x(i*,*j)* is calculated as

$$X_{norm}=\frac{x_{\left(i,j\right)}-mean(X_{j})}{std(X_{j})},$$ (1)

where *X*_*j*_ is the *j*^*th*^ column of matrix *X*. Next, the principal component analysis (PCA) [65] method was applied to achieve dimensionality reduction. PCA simplifies complex high-dimensional data while retaining important trends and patterns. It is a feature selection process that creates new features from a linear combination of initial features. When performing PCA, the main goal is to find an orthonormal set of axes that point in the direction of the maximum covariance matrix for the data. PCA maps *d*-dimensional space data into a new *k*-dimensional subspace, where *k* < *d*. The new *k* variables are referred to as principal components (PCs), where each PC has a maximum variance reflecting the variance that was accounted for in all preceding components. PCA is an unsupervised learning method that provides a very powerful and reliable tool for data analysis. As mentioned above, once specific patterns in the data are identified, the data can be compressed into lower dimensions. Here, the number of PCs was determined by maintaining a variance level greater than 99%. From Fig 4, it can be seen that the first 61 PCs preserved 99% of the total variance. Therefore, the first 61 features were extracted as PCs for AD vs. HC classification. The same procedure was used for the other classification problems.

### Classification methods

In this study, we used three different popular classifiers to evaluate classification performance based on single and combined features.

#### Support vector machine

SVM is a discriminative classifier that is formally defined by a separating hyperplane [13,23,29]. In other words, it is a supervised learning method that uses a training dataset to find an optimal separating hyperplane in an *n*-dimensional space. The optimal hyperplane is one that best separates the two target subject groups. Test subjects are then categorized according to their comparative position, which defines a hyperplane in the *n*-dimensional feature space. In our study, we used the LIBSVM library, which is equipped with a radial basis function (RBF) kernel. An RBF kernel performs better than a linear kernel for a small number of features. A regularization constant *C* and a set of kernel hyperparameters *γ* are the hyperparameters required by the SVM. These parameters are optimized by using a cross-validation (CV) method.

#### Random forest

The RF algorithm is a supervised method that uses an ensemble learning technique for classification [66,67]. It operates by structuring a multitude of decision trees during training and outputting the class that represents the average output of the individual trees during testing. The RF algorithm is typically implemented using the methodology of classification or regression trees (CARTs), where a binary splitting operation recursively partitions trees into homogenous or near-homogenous terminal nodes. A desirable binary split pushes data from a parent node to its child nodes such that the homogeneity in the child nodes is better than that in the parent node. An RF is a group of 100 to 1000 of trees, where each tree is constructed by using bootstrapped samples from the original data. RF trees are different from traditional CARTs because they are constructed non-deterministically according to a two-stage randomization method. The first randomization method is implemented by growing trees using bootstrapped samples from the original data, and the second layer of randomization is introduced at the node level when growing the tree. Rather than splitting a tree node using all variables (for each node in each tree), RF selects a random subset of variables and only those variables are used as predictors to determine the best split for the node. The purpose of this twostep randomization technique is to de-correlate the nodes so that forest ensemble will have low variance and manifest the bagging phenomenon. In our study, we used an RF classifier from the Scikit-learn 0.19.2 Python library.

#### K-Nearest neighbor

KNN is a simple algorithm that belongs to the family of instance-based, competitive learning, and lazy learning algorithms [68,69]. It stores all available labels and classifies new labels according to a similarity measure. For real-valued data, Euclidean distance can be used as a similarity measure. For other types of data, such as categorical or binary data, Hamming distance can be used. KNN makes predictions using a training dataset directly. Predictions are made for a new sample *x* by searching through the entire training dataset for the *K* most similar samples (neighbors) and taking the most common output label for those *K* samples as the label for *x*. In other words, each instance votes for its class and the class with the most votes is taken as the prediction. Class probabilities can be calculated as the normalized frequencies of the samples that belong to each class in the set of the *K* most similar samples. The Scikit-learn 0.19.2 KNN Python library was used in our experiments.

### Statistical analysis

For each group (AD vs. HC, aAD vs. mAD, and HC vs. mAD), we calculated the Cohen’s kappa [70] statistical value, which measures the inter-annotator (rater) agreement for these classification groups. It is a metric unit that compares an experiential accuracy with an expected accuracy (random chance). Cohen’s kappa statistic is used not only to evaluate an individual classifier but also to evaluate classifiers between themselves. Moreover, it considers random chance (agreement between random classifiers), which usually means that it is less deceptive than simply using the accuracy as a metric unit. Classifiers built and assessed on the datasets of a different classes of distributions can be compared more consistently through the kappa statistic (as opposed to merely using the accuracy) because of the scaling technique that is related to the expected accuracy. Cohen’s kappa statistic is also a better indicator of how well a classifier performs across all the instances because a simple percent of accuracy can be tilted if the class distribution is equally skewed. It is defined as

$$k=(p_{0}-p_{e})/(1-p_{e})$$ (2)

where *p*_0_ is the empirical probability of agreement (or observed accuracy) on the label assigned to any sample and *p*_*e*_ is the expected agreement (expected accuracy) when both annotators assign labels randomly. *p*_*e*_ is estimated using a per-annotator empirical prior to the class labels. The kappa statistic value lies between -1 and 1. The maximum value means a complete agreement, whereas zero or lower means worse or chance agreement.

## Experiments and results

In this section, the experiment results obtained through the combined and single features are presented and shown in the tables below. In this study, four class of data were used: AD, aAD, mAD, and HC. The idea of the proposed technique is to combine three extracted features, namely, VBM, CSC, and HV, from SPM12 and Freesurfer to differentiate between AD and other groups. Moreover, we validated our proposed method on three different types of classification problem, i.e., three binary class problems (AD vs. HC, aAD vs. mAD, and HC vs. mAD) as shown in Fig 1. Here, we used three individual features (VBM, CSC, and HV) and a combination of them to classify the three different types of the classification problem. For these cases, we used the NRCD dataset, which is a private dataset. First, we extracted the voxel-based features from each sMR image using SPM12 and then we used Freesurfer to extract the cortical, subcortical, and hippocampus features from each sMR image. Here, we used an early fusion scheme to concatenate the three features (VBM, CSC, and HV) into one. It is a simple method that combines the different modalities of features into a single feature vector, and then we train a classifier on that single feature vector. Moreover, we applied a feature selection technique using PCA, which will select the effective features from the original features and send these selected features to a classifier, to measure the performance of each classification group. In this study, we used three different types of classifier: KNN, SVM, and RF. To obtain unbiased estimates of the performance, we randomly split the set of participants into two groups a training dataset and a testing dataset, at a ratio of 70:30, respectively. In the training dataset, a five-fold stratified cross-validation technique was applied to obtain the optimal hyperparameter values for the cost function, *C*, and *γ* for SVM; Max_features, Criterion, Max_depth, and optimal hyperparameter values for RF; and n_neighbors optimal hypermeter value for KNN. These optimal hyperparameter values were calculated by using a grid-search CV library function from Scikit-learn 0.19.2 and also by applying a five-fold stratified cross-validation method on the training set. For each method, the obtained optimized hyperparameters value was then used to train the classifier using the training data, and then the performance of each resulting classifier was evaluated using 30% of the testing dataset. In this way, we achieved unbiased estimates for each classification group. To evaluate whether each classification group had an inter-annotator (rater) agreement or not, we calculated the Cohen’s kappa statistic index. Moreover, we plotted the receiver operating characteristic (ROC) curves then calculated the area under the curve value for each classification problem. The AUC was invariant to the class distribution, which was an advantage since the number of control subjects was larger than the number of AD patients. We also calculated the classification accuracy, sensitivity, precision, specificity, and F1 values for the classification groups. We repeated the cross-validation procedure five times to obtain a more reliable cross-validation error and extracted the mean AUC value for each classification group.

Our experiments were conducted in two stages. In the first stage, only individual features were selected, such as VBM-extracted ROI volumes, CSC-extracted feature volumes, and HV-extracted features. These features were then fed into the classifiers one at a time to measure the individual feature performance. In the second stage, combinations of all features (also called single feature vector) were applied to individual classifiers to measure the classification performance. Moreover, a 95% confidence interval for ACC was estimated according to multiple classification runs. To evaluate whether each technique performed significantly better than a random classifier, we used McNemar’s chi-squared test with a significance threshold of 0.05, which is a typical benchmark value. This test analyzes the differences between proportions in paired observations. It was used to assess the differences between the proportions of correctly classified subjects (i.e., ACC). The corresponding contingency chart is provided in Table 3. We also used McNemar’s chi-squared test to evaluate the differences between the proportions of incorrectly classified subjects. All experiments were conducted on a computer running Ubuntu Linux version 16.04 with Python version 3.6.

**Table 3:** Contingency table for a McNemar’s test.

Group	Group 2: Correctly classified	Group2: Misclassified
**Group 1: Correctly Classified**	AA	BB
**Group 1: Misclassified**	CC	DD

### AD vs. HC

We calculated the statistical values that represent the significance levels of clusters in the activation map as shown in Table 4 after comparing the results of the statistical two-sample *t*-tests for the AD vs. HC group. Table 4 specifies the main affected area distributed in the AD vs. HC group, and an achieved voxel clusters with detailed information including its peak coordinates in the form of Montreal Neurological Institute Space, cluster-level in *p*-value, and the peak intensity in *T*-value of each cluster, which is given below. We used an uncorrelated threshold value of *P*_*uncorrected*_ ≤ 0.001 at the voxel level, FDR value of *P*_*FDR*_ = 0.05, and an FWER value of *P*_*FWER*_ = 0.05 at the cluster level to perform a bias correction for multiple comparisons. An ROI binary mask was created from the five selected clusters and later GM volume was extracted from the two groups of images (AD and HC). The minimum cluster size in this study was kept as 200 voxels because while applying two-sample *t*-test in this group, we find a large number of differences in their GM region while comparing them. Here, each cluster contains more than 200 adjacency voxels that show the significant variation of those diffusion parameters. The selected significant voxels are displayed with their *T*-values. The ROI was defined by comprising the suprathreshold intensity voxels. Moreover, the integral of suprathreshold intensities within a cluster naturally combines both signal extent and signal intensity. Hence, from Table 4, we can say that for AD vs HC group, both suprathreshold intensities (positive and negative) has shown the significant affected region while overlapping AD subject images over HC patient images for extracting GM difference region as a cluster which was shown by their *T*-value. The ascending peak intensity (*T*-value) is shown from the darkness to brightness.

**Table 4:** Cluster information (AD vs. HC).

Cluster	Number of voxels	Peak MNI coordinates			Peak MNI coordinate region	Peak intensity (T-Value)
		X	Y	Z		
**Cluster 1**	2266	-25.5	-16.5	-35.5	Hippocampus_L	14.3469
**Cluster 2**	1757	16.5	24	18	Right Cerebrum	-5.0396
**Cluster 3**	261	-21	-63	16.5	Frontal lobe	-4.3777
**Cluster 4**	619	-28.5	-63	16.5	Left Cerebrum	-4.9033
**Cluster 5**	337	31.5	-55.5	25.5	Occipital_Inf_R	-4.1527

**Fig 5:**
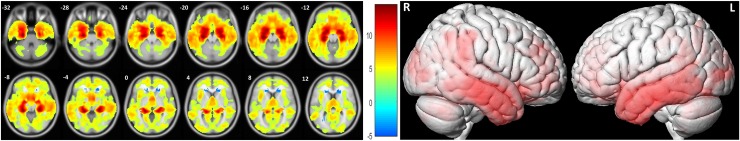
Results showing significant GM differences between the AD and HC groups.

As shown in Fig 5, the left brain shows significant differences in GM probability between the AD and the HC group. The dark region of positive correlation also shows differences in the hippocampus region, including significant atrophy in the AD group as compared to the HC group. Fig 5 also shows that the left hemisphere of the hippocampus (2266 voxels) region has a significant GM volume loss when comparing the AD group with the HC group, and their peak intensity (*T*-value) value is 14.3469.

### aAD vs. mAD

**Fig 6:**
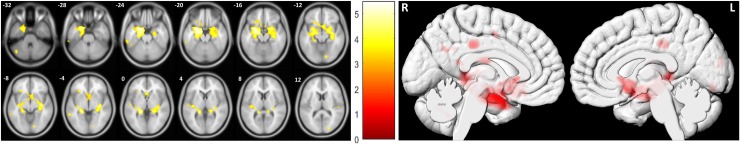
Results showing significant GM differences between the aAD and mAD groups.

Table 5 shows the main affected area distributed in the aAD vs. mAD group, and an achieved voxel clusters with detailed information including its peak coordinates in the form of Montreal Neurological Institute Space, cluster-level in *p*-value, and the peak intensity in *T*-value of each cluster, which are given below. For the aAD vs. mAD group, we used an uncorrelated threshold value of *P*_*uncorrected*_ ≤ 0.001 at the voxel level, FDR value of *P*_*FDR*_ = 0.05, and an FWER value of *P*_*FWER*_ = 0.05 at the cluster level, to perform a bias correction for multiple comparisons. The minimum cluster size in this study was kept as 100 voxels because while applying two-sample *t*-test in this group, we didn’t find much difference in their GM region while comparing them. The obtained cluster information is listed in Table 5. Here, each cluster contains more than 100 adjacency voxels that show the significant variation of those diffusion parameters. The selected significant voxels are displayed with their *T*-values. From Table 5, we can say that for aAD and mAD group, only negative suprathreshold intensity has shown the significant affected region while overlapping aAD subject images over mAD patient images for extracting GM difference region as a cluster, which was shown by their *T*-value, whereas no significant region is found on positive suprathreshold intensity. That is why there is no region for positive peak intensity, and also because of small GM differences in this region, their peak intensity value looks similar. The ascending peak intensity (*T*-value) is shown from the darkness to brightness. We selected nine clusters to construct an ROI brain mask. Fig 6 shows that the left hemisphere of the Para_hippocampus (3560 voxels) and occipital (191 voxels) regions has a significant amount of GM volume loss when comparing the aAD group with the mAD group, and their peak intensity (*T*-value) are -5.4652, and -4.788, respectively.

**Table 5:** Cluster information (aAD vs. mAD).

Cluster	Number of voxels	Peak MNI coordinates			Peak MNI coordinate region	Peak intensity (T-Value)
		X	Y	Z		
**Cluster 1**	232	-33	-55.5	-40.5	Cerebellum Posterior Lobe	-3.8098
**Cluster 2**	3560	34.5	-19.5	-4.5	Para_hippocampus_L	-5.4652
**Cluster 3**	230	-60	-34.4	-22.5	Temporal_Pole_Sup_L	-3.6969
**Cluster 4**	129	13.5	-82.5	-9	Lingual	-4.2339
**Cluster 5**	191	21	-90	15	Occipital_Sup_R	-4.788
**Cluster 6**	285	-9	-16.5	22.5	Cerebro-Spinal Fluid	-4.2348
**Cluster 7**	151	-10.5	-57	30	Precuneus	-4.1502
**Cluster 8**	306	-4.5	-28.5	37.5	Cingulum	-4.3694
**Cluster 9**	117	-13.5	-3	49.5	Medial Frontal Gyrus	-4.6933

### HC vs. mAD

Table 6 shows the on-line table for main affected area distributed in the HC vs. mAD group, and an achieved voxel clusters with detailed information, including its peak coordinates in the form of Montreal Neurological Institute Space, cluster-level in p-value, and the peak intensity in *T*-value of each cluster are given below.

**Table 6:** Cluster information (HC vs. mAD).

Cluster	Number of voxels	Peak MNI coordinates			Peak MNI coordinate region	Peak intensity (T-Value)
		X	Y	Z		
**Cluster 1**	3337	-27	3	-21	Hippocampus_R	-5.4644
**Cluster 2**	509	-48	-42	-10.5	Fusiform Gyrus	-3.8461
**Cluster 3**	222	7.5	13.5	-12	Olfactory_R	-3.5835
**Cluster 4**	331	21	49.5	12	Medial Frontal Gyrus	-3.8868
**Cluster 5**	313	28.5	-19.5	21	Precentral Gyrus	-4.1624
**Cluster 6**	4541	27	-54	22.5	Right Cerebrum	3.5911
**Cluster 7**	755	-9	-60	33	Precuneus_L	-4.641
**Cluster 8**	765	-7.5	13.5	36	Sub-Gyral_L	-4.2398

**Fig 7:**
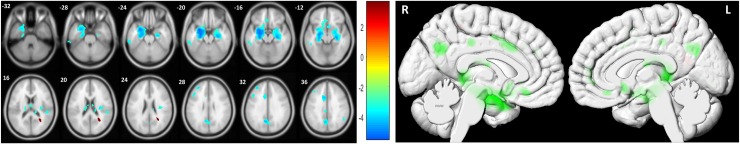
Results showing significant GM differences between the HC and mAD groups.

**Fig 8:**
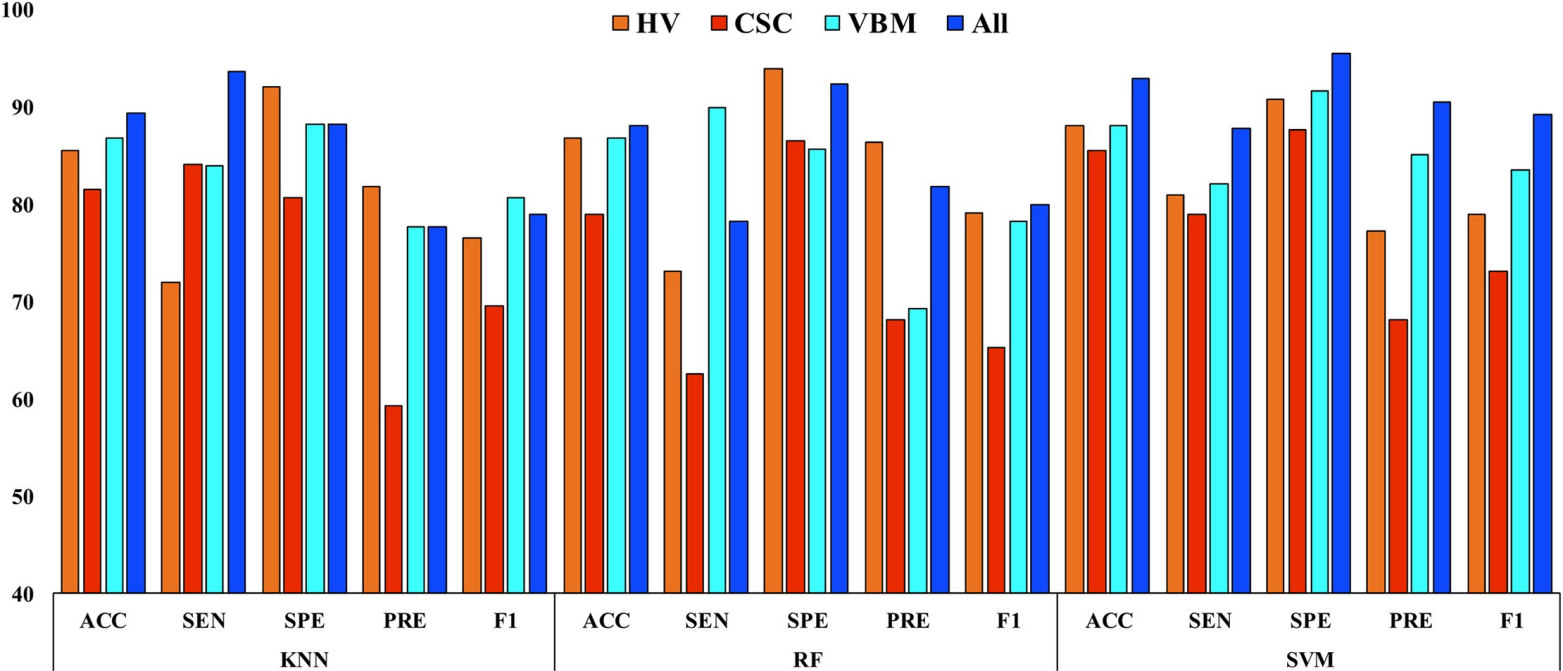
Classification results for AD vs. HC.

**Fig 9:**
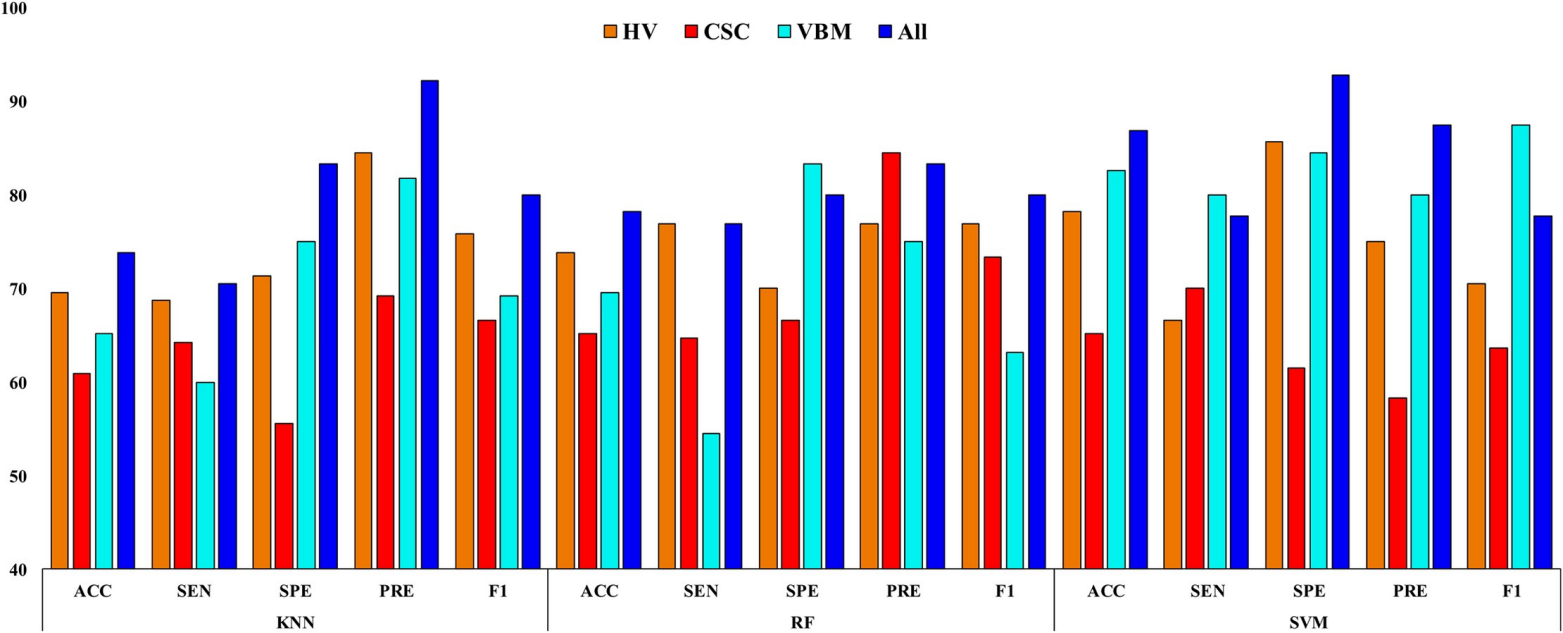
Classification results for aAD vs. mAD.

**Fig 10:**
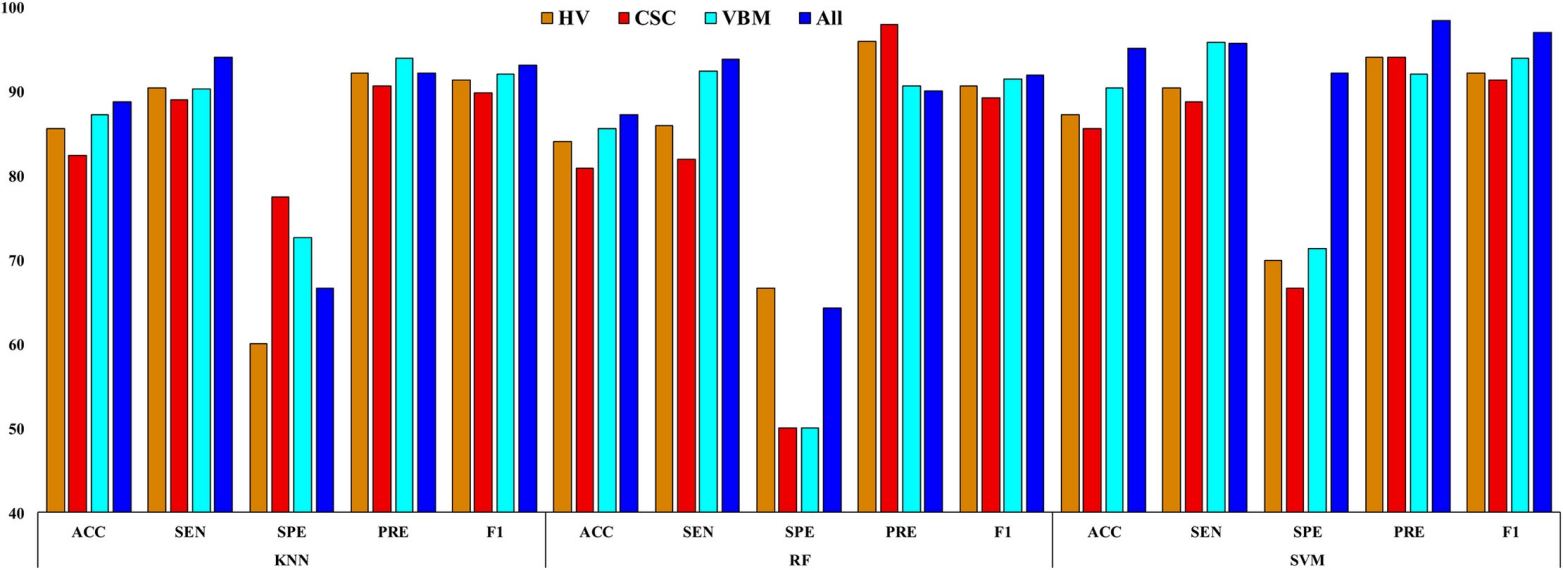
Classification results for HC vs. mAD.

For the HC vs. mAD group, we applied uncorrected threshold value of *P*_*uncorrected*_ ≤ 0.001 at the voxel level, FDR value of *P*_*FDR*_ = 0.05, and an FWER value of *P*_*FWER*_ = 0.05 at the cluster level to generate eight clusters. The obtained cluster information is listed in Table 6. The minimum cluster size in this study was kept as 300 voxels because while applying two-sample *t*-test in this group we found a large number of differences in their GM regions while comparing them. Here, each cluster contains more than 300 adjacency voxels that show the significant variation of those diffusion parameters. The selected significant voxels are displayed with their *t*-values. From Table 6, we can say that for HC vs. mAD group, both suprathreshold intensities (positive and negative) has shown the significant affected region while overlapping HC subject images over mAD patient images for extracting GM difference region as a cluster which was shown by their *T*-value. The ascending peak intensity (*T*-value) is shown from the darkness to brightness. Fig 7 shows that the right hemisphere of the Cerebrum and hippocampus (4541 voxels, and 3337 voxels) region has a significant GM volume loss when comparing the HC group with the mAD group, and their peak intensity (*T*-value) is 3.5911, and -5.4644, respectively. Tables 7–9 and Figs 8–10 show the classification result for AD vs. HC, aAD vs. mAD, and HC vs. mAD.

**Table 7:** Classification results for AD vs. HC.

Groups	Classifier		Performance measure						McNemar’s test
		AUC	ACC	SEN	SPE	PRE	F1	Cohen’s	
**HV**	KNN	85.87	85.52	72	92.15	81.81	76.59	0.6812	P<0.000001
	RF	87.19	86.84	73.07	94	86.36	79.16	0.7077	P<0.000001
	SVM	88.67	88.15	80.95	90.9	77.27	79.06	0.7123	P<0.000001
**CSC**	KNN	80.26	81.57	84.21	80.7	59.25	69.56	0.6694	P<0.000001
	RF	78.15	78.94	62.5	86.53	68.18	65.21	0.6245	P<0.000001
	SVM	86.10	85.52	78.94	87.71	68.81	73.17	0.6894	P<0.000001
**VBM**	KNN	87.19	86.84	84	88.23	77.77	80.26	0.7202	P<0.000001
	RF	86.77	86.84	90	85.71	69.23	78.26	0.6973	P<0.000001
	SVM	89.56	88.15	82.14	91.66	85.15	83.63	0.7367	P<0.000001
**Combined (ALL)**	KNN	90.54	89.47	93.75	88.33	77.77	78.94	0.7565	P<0.000001
	RF	90.43	88.15	78.26	92.45	81.81	80	0.7237	P<0.000001
	SVM	93.93	93.06	87.87	95.58	90.62	89.23	0.9056	P<0.000001

**Table 8:** Classification results for aAD vs. mAD.

Groups	Classifier		Performance measure						McNemar’s test
		AUC	ACC	SEN	SPE	PRE	F1	Cohen’s	
**HV**	KNN	70.55	69.56	68.75	71.42	84.61	75.86	0.6155	P = 0.22
	RF	73.29	73.91	76.92	70	76.92	76.92	0.6202	P = 0.17
	SVM	79.45	78.26	66.66	85.71	75	70.58	0.7078	P = 0.0064
**CSC**	KNN	61.27	60.86	64.28	55.55	69.23	66.66	0.5589	P = 0.5
	RF	67.24	65.21	64.7	66.66	84.61	73.34	0.5645	P = 0.34
	SVM	65.42	65.21	70	61.53	58.33	63.63	0.5483	P = 0.29
**VBM**	KNN	66.47	65.21	60	75	81.81	69.23	0.5888	P = 0.14
	RF	70.44	69.56	54.54	83.33	75	63.15	0.6345	P = 0.019
	SVM	83.82	82.6	80	84.61	80	87.48	0.7154	P = 0.011
**Combined (ALL)**	KNN	74.69	73.91	70.58	83.33	92.3	80	0.6988	P = 0.10
	RF	79.99	78.26	76.92	80	83.33	80	0.7244	P = 0.054
	SVM	87.08	86.95	77.77	92.85	87.5	77.77	0.7606	P = 0.0009

**Table 9:** Classification results for HC vs. mAD.

Groups	Classifier		Performance measure						McNemar’s test
		AUC	ACC	SEN	SPE	PRE	F1	Cohen’s	
**HV**	KNN	87.87	85.71	90.56	60	92.3	91.42	0.7746	P = 0.37
	RF	83.21	84.12	85.96	66.66	96.07	90.74	0.7477	P = 0.34
	SVM	87.12	87.3	90.56	70	94.11	92.3	0.7881	P = 0.17
**CSC**	KNN	83.37	82.52	89.09	77.5	90.74	89.9	0.7699	P = 0.36
	RF	83.45	80.95	81.96	50	98.03	89.28	0.7041	P = 0.18
	SVM	86.99	85.71	88.88	66.66	94.11	91.42	0.7237	P = 0.25
**VBM**	KNN	89.47	87.3	90.38	72.72	94	92.15	0.7745	P = 0.11
	RF	86.54	85.71	92.45	50	90.74	91.58	0.7345	P = 0.5
	SVM	81.45	90.47	95.91	71.42	92.15	94	0.8214	P = 0.089
**Combined (ALL)**	KNN	89.57	88.88	94.11	66.66	92.3	93.2	0.7645	P = 0.19
	RF	88.56	87.3	93.87	64.28	90.19	92	0.7589	P = 0.21
	SVM	95.83	95.23	95.77	92.30	98.50	97.14	0.9468	P = 0.016

**Fig 11:**
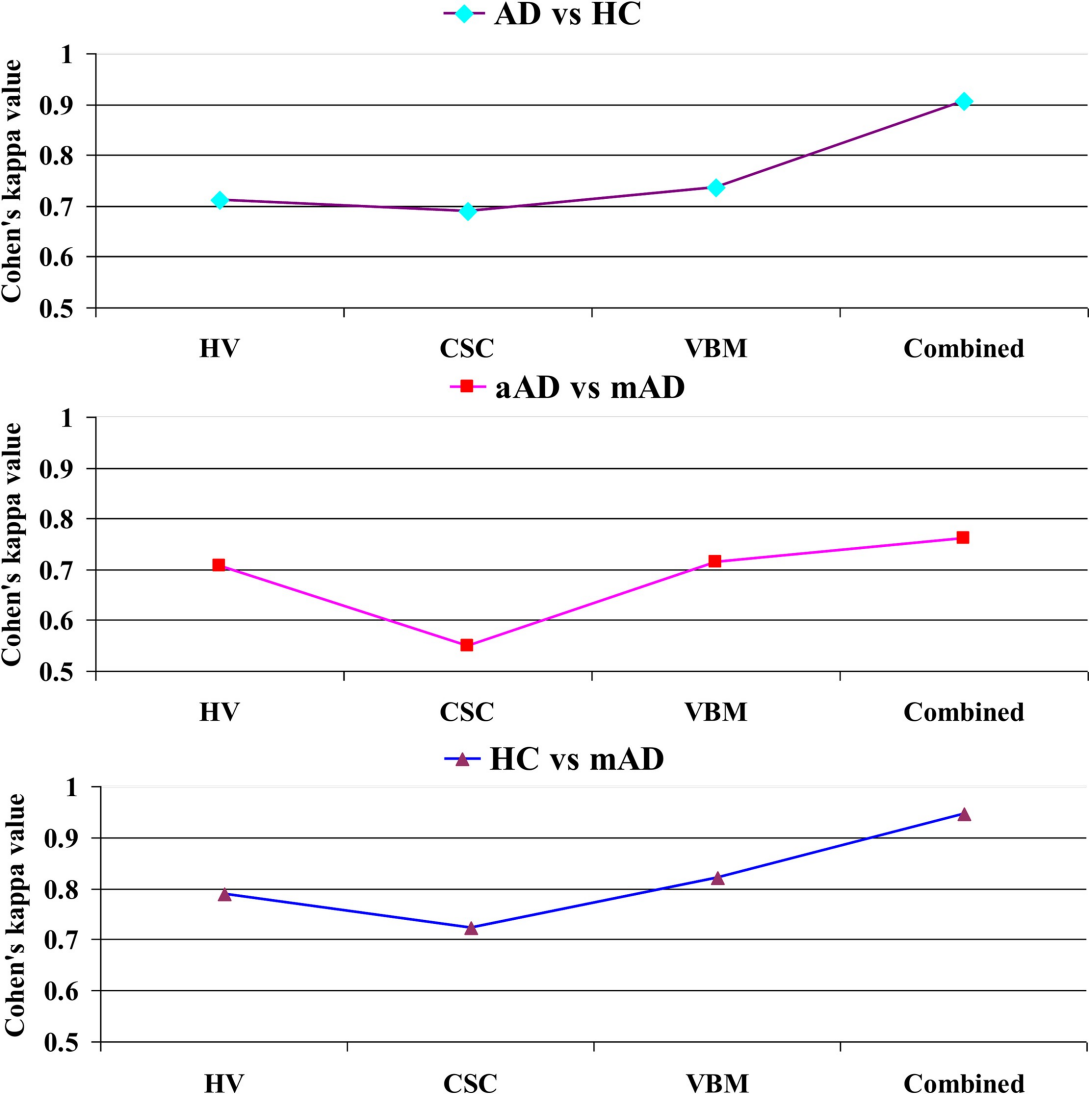
Cohen’s kappa score graph for (a) AD vs. HC, (b) aAD vs. mAD, (c) HC vs. mAD using SVM classifier.

Fig 11 shows Cohen’s kappa statistic graph for three classification groups (AD vs. HC, aAD vs. mAD, and HC vs. mAD). From this graph, we can see that our proposed method achieved a good level of agreement within each group when classifying the AD vs. HC, aAD vs. mAD, and HC vs. mAD groups using the combined features. In this study, the combined features with the SVM classifier achieved Cohen’s kappa values of 0.9056, 0.7606, and 0.9468, which are close to 1, for AD vs. HC, aAD vs. mAD, and HC vs. mAD, respectively. As can be seen from Tables 7–9, the SVM classifier with the combined features achieved a higher level of the agreement compared to that with individual features.

**Fig 12:**
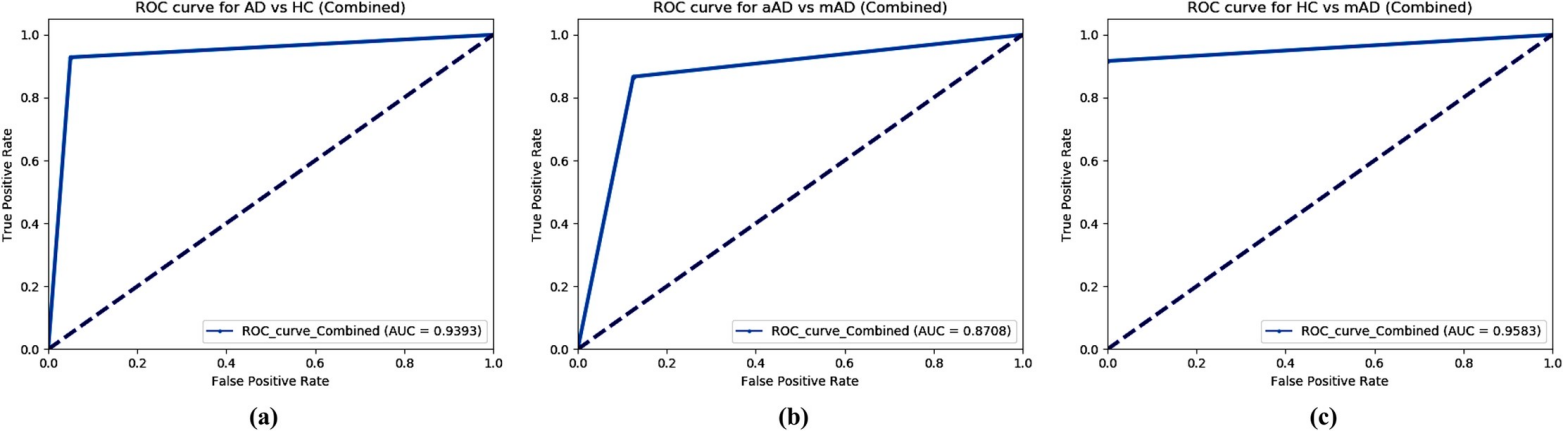
AUC graph for (a) AD vs. HC, (b) aAD vs. mAD, (c) HC vs. mAD groups using SVM classifier.

Here, Fig 12 shows the AUC curve for the AD vs. HC, aAD vs. mAD, and HC vs. mAD classification groups. Moreover, for the AD vs. HC group, our proposed model achieved an AUC of 93.93%, which indicates that it performed very well when distinguishing AD subjects from HC subjects using combined features. Moreover, for the aAD vs. mAD group, our proposed model correctly classified the converted patients when compared to the stable patients, with an AUC of 87.08%. Likewise, our proposed model achieved an AUC of 95.83% for the HC vs. mAD group. Overall, for all classification methods, our proposed model performed well and its probabilities from the positive classes were well separated from the negative classes. From Tables 7–9, we can see that the SVM classifier with a combined features achieved better AUC result compared to that with individual features.

## Comparison to State-of-the-Art methods

The NRCD dataset is not available publicly for research purposes. Therefore, to compare our method with other state-of-the-art methods, we applied our proposed method on the ADNI public dataset, which can be downloaded from http://adni.loni.usc.edu/. The ADNI data center was established in 2003 as a public-private corporation with aid from several groups within the organization, including the National Institute of Aging and National Institute of Biomedical Imaging and Bioengineering, as well as several non-profit organizations and private pharmaceutical companies. The primary objective of the ADNI dataset has is to determine whether serial MRI, PET, various biological biomarkers, and clinical and neuropsychological tests can be combined to assess the progression of MCI and early AD symptoms. For up-to-date information, please visit www.adni-info.org.

For this study, a total of 163 sMR images (belonging to one of the AD, MCIc (MCI converted to AD, who had converted to AD within a 24-month time period), MCIs (stable MCI, who had not converted to AD within a 24-month time period), and HC groups) were acquired from the ADNI dataset. Patients with MCI and HC subjects were chosen randomly from a database of participants in longitudinal studies who were monitored for at least 24 months. All subjects underwent several neuropsychological examinations to produce several clinical characteristic indicators in combination with MMSE results, functional assessment questionnaire scores, and clinical dementia ratios. Table 10 shows the demographic information for all subjects.

**Table 10:** Subjects demographics information.

Group	AD	MCIc	MCIs	HC
**No. of subjects**	30	50	43	40
**Male/Female**	13/17	33/17	25/18	21/19
**Age (SD)**	77.4 (6.31)	76.92 (7.35)	73.023 (6.14)	78.525 (5.30)
**MMSE (SD)**	18.97* (5.64)	25.60* (3.61)	26.27* (2.93)	28.99* (1.52)
**FAQ (SD)**	20.30* (6.75)	7.52* (6.78)	6.09* (6.44)	0.50* (1.78)

*Indicates a statistically significant difference from all other groups

Both male and female subjects were included in our experiments. All sMRI scans used in this study were acquired from 1.5-T MRI scanners. First, we extracted voxel-based features using SPM12 and then we extracted cortical, subcortical, and left and right hemisphere hippocampus features from each sMR image using Freesurfer. Here, we followed the same procedures as those for the NRCD dataset. For the classification between different groups, we chose the same classifier as that for the NRCD dataset. Table 11 shows the obtained result for the AD vs. HC group. It can be seen from below table that our proposed method achieved a significantly better *p*-value than the conventional *p*-value threshold of 0.05. Likewise, Tables 12 and 13 show the results for the MCIc vs. MCIs and the HC vs. MCIc classification, where some groups yielded a significantly better *p*-value than the conventional *p*-value of 0.05.

**Table 11:** Classification results for an AD vs. HC.

Groups	Classifier		Performance measure						McNemar’s test
		AUC	ACC	SEN	SPE	PRE	F1	Cohen’s	
**HV**	KNN	94.44	90.47	75	100	100	85.71	0.9114	P = 0.00012
	RF	90.47	92.85	88.88	94.73	90.9	89.01	0.8995	P = 0.00003
	SVM	96.97	96.42	100	92.85	93.33	96.55	0.9245	P = 0.00007
**CSC**	KNN	91.67	89.28	100	85	72.72	84.21	0.8775	P = 0.00012
	RF	87.65	85.71	72.72	94.11	88.88	79.99	0.7956	P = 0.00117
	SVM	93.45	92.85	100	89.47	81.81	90	0.8563	P = 0.00036
**VBM**	KNN	93.33	82.14	75	85	66.66	70.58	0.7879	P = 0.00128
	RF	88.57	89.28	90	88.88	81.72	82.25	0.8134	P = 0.00065
	SVM	95.78	95.23	100	92.85	87.5	93.33	0.8987	P = 0.00091
**Combined (ALL)**	KNN	95.83	95.23	100	93.75	83.83	90.9	0.9041	P = 0.00025
	RF	91.37	92.85	90.9	94.11	89.41	85.71	0.8978	P = 0.00013
	SVM	97.56	96.42	93.8	100	100	96.77	0.9465	P = 0.00001

**Table 12:** Classification results for MCIc vs. MCIs.

Groups	Classifier		Performance measure						McNemar’s test
		AUC	ACC	SEN	SPE	PRE	F1	Cohen’s	
**HV**	KNN	86.15	86.48	85	88.23	89.47	87.17	0.7157	P = 0.0022
	RF	93.57	94.59	100	90	89.47	94.44	0.8679	P = 0.0002
	SVM	91.58	91.89	94.44	91.66	89.47	91.89	0.8235	P = 0.0036
**CSC**	KNN	82.38	81.08	90.47	68.75	79.16	84.44	0.6345	P = 0.1050
	RF	80.54	78.37	94.44	63.15	70.83	80.95	0.6834	P = 0.0351
	SVM	87.47	86.48	88.88	84.21	87.78	86.78	0.7857	P = 0.0384
**VBM**	KNN	84.38	86.84	79.16	100	100	88.37	0.7535	P = 0.00006
	RF	92.54	92.1	91.3	93.33	95.45	93.33	0.8988	P = 0.00003
	SVM	89.88	89.47	86.2	100	100	92.59	0.8545	P = 0.00012
**Combined (ALL)**	KNN	96.15	96.42	100	92.3	93.75	96.77	0.9278	P = 0.00317
	RF	82.78	81.89	85.71	100	100	92.3	0.7453	P = 0.00001
	SVM	96.89	97.36	96.15	100	100	98.03	0.9345	P = 0.000001

**Table 13:** Classification results for HC vs. MCIc.

Groups	Classifier		Performance measure						McNemar’s test
		AUC	ACC	SEN	SPE	PRE	F1	Cohen’s	
**HV**	KNN	71.98	72.22	66.66	80	82.35	73.68	0.6345	P = 0.0192
	RF	91.53	90.90	88.88	93.33	94.11	91.42	0.8878	P = 0.0004
	SVM	92.45	91.89	88.88	94.73	94.11	91.42	0.8723	P = 0.0033
**CSC**	KNN	73.21	74.07	61.11	100	100	75.86	0.6544	P = 0.0019
	RF	80.88	80.55	70.58	89.47	85.71	77.41	0.7252	P = 0.0036
	SVM	84.45	83.33	70	100	100	82.35	0.7945	P = 0.0045
**VBM**	KNN	81.98	81.48	71.42	92.3	90.9	80	0.7216	P = 0.0063
	RF	87.65	88.88	85.71	90.9	85.71	82.67	0.8144	P = 0.0011
	SVM	91.45	89.47	85.71	91.66	82.23	88.88	0.8365	P = 0.0038
**Combined (ALL)**	KNN	72.16	70.37	64.7	80	84.61	73.33	0.6456	P = 0.0546
	RF	87.67	86.11	73.68	100	100	84.84	0.8465	P = 0.0046
	SVM	95.43	94.73	90.90	100	100	95.23	0.9260	P = 0.000015

The proposed method achieved better result for all three classification groups. For the AD vs. HC group, the combined features with the SVM classifier achieved an AUC of 97.56%, and an ACC of 96.42% compared to the best individual feature output, which was obtained by HV features using the SVM classifier, and the obtained Cohen’s kappa value for this group was 0.9465, which is close to 1, and also showed a high level of agreement between these two groups. Moreover, for the MCIs vs. MCIc group, our proposed method with combined features achieved better results (AUC of 96.89%, and ACC of 97.36%) and the obtained Cohen’s kappa value for this group was around 0.9345, which is close to 1, and demonstrate a high-level of concurrence between the MCIs and the MCIc group. Likewise, for the HC vs. MCIc group, our proposed technique with combined features obtained better results (AUC of 95.43%, and ACC of 94.73%) and the obtained Cohen’s kappa value for this group was 0.9260, which is close to 1, and demonstrate a high-level of concurrence between the HC and the MCIc group.

Recently, several studies have reported their classification results for distinguishing AD patients from HC individuals according to the MRI dataset. Zhang et al. [23] performed a multimodal classification of AD based on a combination of MRI, CSF, and PET data. By using MRI data, they achieved an ACC of 86.2% for the AD vs. HC classification. By combining all the aforementioned biomarkers, they achieved a higher ACC of 93.2%. Westman et al. [27] reported an ACC of 87% when using MRI data and an increased ACC to 91% when combining MRI data with CSF measures. Lama et al. [32] adopted Freesurfer to compute the CTH and volumetric measurements. Using an extreme learning machine classifier, they achieved an ACC of 77.88%. Cuingnet et al. [13] compared 10 widely used methods using the ADNI dataset. They used three different techniques to extract features from the brain: VBM, CTH, and HV. They reported an SEN of 81% and an SPE of 95% as the best performance measures when using the Voxel-Direct-D-gm method. Wolz et al. [29] used a multi-method technique for the early detection of AD. They considered four types of features, namely, HV, CTH, TBM, and manifold-based learning, which they combined together to achieve an ACC of 89% using linear discriminant analysis and 86% using an SVM classifier. Jha et al. [71] proposed a technique using complex dual-tree wavelet PC features and an extreme learning machine as a classifier. For the ADNI dataset, their method achieved an ACC of 90.26%. Beheshti et al. [72] proposed a CAD system composed of four systematic stages for analyzing global and local differences in the GM of AD patients compared to that of HC individuals using a VBM technique. They used seven different feature ranking methods, and with their Fisher’s criterion as a stopping criterion method they achieved an ACC of 92.48% for the AD vs. HC classification. Comparison results of the proposed technique with other published classification methods (using different biomarkers) on the ADNI dataset are provided in Table 14. The table shows that the performance of the proposed method using combined features from sMRI data (VBM+CSC+HV) is superior or comparable to that of other methods reported in the literature.

**Table 14:** Comparison between the proposed method and existing methods.

Approach	Year	Dataset	Modalities	AD/HC	ACC	SEN	SPE
**Zhang at al. [23]**	2011	ADNI	MRI	51/52	86.2	86	86.3
**Westman et al. [27]**	2012	ADNI	MRI	96/111	87	83.30	90.10
**Lama et al. [32]**	2011	ADNI	MRI	70/70	77.88	77.88	68.85
**Cuingnet et al. [13]**	2011	ADNI	MRI	162/137	-	81	95
**Wolz et al. [29]**	2011	ADNI	MRI	198/231	89	93	85
**Jha et al. [71]**	2017	ADNI	MRI	86/86	90.26	90.26	90.27
**Beheshti and Demirel et al. [72]**	2016	ADNI	MRI	130/130	92.48	91.07	93.89
**Proposed Method**	2019	ADNI	MRI	30/40	**96.42**	**93.8**	**100**
	2019	NRCD	MRI	81/171	**93.06**	**87.87**	**95.58**

## Discussion

In this experiment, we evaluated the automatic diagnostic capabilities of three structural MRI features (VBM, CSC, and HV), both individually and using combined features, for 326 subjects from the NRCD dataset. To the best of our knowledge, this is the first study wherein VBM outputs had been combined with CSC and HV for the classification of AD or MCI. From Tables 7–9, we can be seen that, the VBM individual feature achieved better result than those of other individual features. However, combining all features into one improved the classification performance for all classification groups. These results show that using a combination of various sMRI-based features can improve classification accuracy compared to using only a single feature and can produce a more powerful and steady classifier. To assess and compare the performances of each technique, we performed three classification tests: AD vs. HC, aAD vs. mAD, and HC vs. mAD. Here, we applied three different types of classifier: KNN, SVM, and RF. To obtain unbiased estimates of the performance, we randomly split the set of participants into two groups a training dataset and testing dataset, at a ratio of 70:30, respectively. In the training set, a five-fold stratified CV method was applied to obtain optimal optimized hyperparameter values for the cost function, *C*, and *γ* for SVM; Max_features, Criterion, Max_depth, and optimal hyperparameter values for RF; and n_neighbors optimal hypermeter value for KNN. These optimal hyperparameter values were decided by using a gridsearch CV library function from Scikit-learn 0.19.2 and also by applying a five-fold stratified cross-validation method on the training set. For each method, the obtained optimized hyperparameter value was then used to train the classifier using the training dataset and then the performance of each resulting classifier was evaluated using the remaining 30% of the testing dataset. In this way, we obtained unbiased estimates for each classification group.

### AD vs. HC

The classification results for an AD vs. HC are summarized in Table 7 and are presented in Fig 8. For each case, the dataset was separated into two subsets with a 70:30 ratio. All methods obtained a significantly better *p*-value than the conventional *p-*value of 0.05. The underlying assumption was that there were a significant dissimilarities between these two groups. In our study, the SVM classifier achieved the best results with an AUC of 93.93%, ACC of 93.06%, SEN of 87.87%, SPE of 95.58%, PRE of 90.62%, and F1 of 89.23% for this classification group. Moreover, the obtained Cohen’s kappa value for this group was 0.9056, which is close to 1. This shows that, our proposed model using combined features achieved a high level of agreement between these two groups.

### aAD vs. mAD

The classification results for aAD vs. mAD are summarized in Table 8 and are presented in Fig 9. The SVM classifier with combined features obtained a significantly better *p*-value (0.0009) than the conventional *p*-value of 0.05. It also achieved an AUC of 87.08%, ACC of 86.95%, SEN of 77.77%, SPE of 92.85%, PRE of 87.5%, and F1 of 77.77% for this classification group. Moreover, the obtained Cohen’s kappa value for this group was 0.7606, which is close to 1. This shows that our proposed model achieved a high level of agreement between these two groups.

### HC vs. mAD

The classification results for a HC vs. mAD are summarized in a Table 9 and are presented in Fig 10. The SVM classifier with combined features obtained a significantly better *p*-value (0.016) than the conventional *p*-value of 0.05. It also achieved an AUC of 95.83%, ACC of 95.23%, SEN of 95.27%, SPE of 92.30%, PRE of 98.50%, and F1 of 97.14% for this classification group. Moreover, the obtained Cohen’s kappa value for this group was 0.9468, which is close to 1. This shows that our proposed model achieved a high level of agreement between these two groups.

From Tables 7–9 and Figs 8–10, it can be seen that the AUC and ACC values improved significantly when applying multiple features, compared to using individual features. All the classification methods in this study achieved good AUC and ACC values, and the SVM classifier performed significantly better than the KNN and RF classifiers for distinguishing AD or mAD patients from HC individuals. Still, our study should be considered as a preliminary proof-of-concept study. It proposes a promising approach for the future translation of neuroimaging into patient benefit. This approach requires replication and validation in larger samples; however, it provides initial evidence of a rapid and accessible methodology that could potentially aid clinical decisions.

## Conclusions

In this paper, a novel feature fusion technique was proposed to improve the classification accuracy of the AD, aAD, mAD, and HC groups. First, we preprocessed sMR images and used CAT12, which is integrated with the SPM12 software, for the extraction of specific ROIs. We then used Freesurfer to extract CSC and HV features from 326 subjects. Finally, we merged these three types of features linearly and used the combined features for the early prediction of AD. We found that the combination of morphometric features with cortical and HV features performed better than any individual features. Additionally, the proposed method achieved an AUC and ACC of 87.08% and 86.95%, respectively, for the aAD vs. mAD classification group (NRCD dataset) and 96.89% and 97.36%, respectively, for the MCIc vs. MCIs classification (ADNI dataset). The *p*-value obtained by McNemar’s chi-squared test for aAD vs. mAD and MCIs vs. MCIc group was less than the conventional *p*-value threshold of 0.05. The obtained Cohen’s kappa value for aAD vs. mAD was 0.7606 for the NRCD dataset and for MCI vs MCIc was 0.9345 for the ADNI dataset, which is close to 1. We can say that this classification group achieved a high level of agreement with each other. The proposed method main advantages are as follows. First, it uses combined features that can be extracted from a sMRI modality. Second, it uses three different classifiers to achieve the best possible AUC and ACC values. We performed several evaluation experiments on the private NRCD dataset using optimized hyperparameters. The test results were analyzed and presented in this paper, and they showed the efficiency of the proposed model for improving classification performance.

The method proposed in this study performed better in every classification group. However, it still has some drawbacks as it was only tested on a relatively small dataset. In the future, we will apply the proposed model to a large publicly available datasets. Additionally, we also plan to examine different imaging modalities, such as PET and functional MRI, for the early detection of AD.

## References

[1] Braak H, Braak E, Bohl J, Bratzke H. Evolution of Alzheimer’s disease related cortical lesions In: Gertz H-J, Th Arendt, editors. Alzheimer’s Disease—From Basic Research to Clinical Applications. Vienna: Springer Vienna; 1998 pp. 97–106. 10.1007/978-3-7091-7508-8_9 9850918

[2] Bain LJ, Jedrziewski K, Morrison-Bogorad M, Albert M, Cotman C, Hendrie H, et al Healthy brain aging: A meeting report from the Sylvan M. Cohen Annual Retreat of the University of Pennsylvania Institute on Aging. Alzheimer’s & Dementia. 2008;4: 443–446. 10.1016/j.jalz.2008.08.006 18945646 PMC2592556

[3] Brookmeyer R, Johnson E, Ziegler-Graham K, Arrighi HM. Forecasting the global burden of Alzheimer’s disease. Alzheimer’s & Dementia. 2007;3: 186–191. 10.1016/j.jalz.2007.04.381 19595937

[4] Wattmo C, Londos E, Minthon L. Risk Factors That Affect Life Expectancy in Alzheimer’s Disease: A 15-Year Follow-Up. Dementia and Geriatric Cognitive Disorders. 2014;38: 286–299. 10.1159/000362926 24992891

[5] Dubois B, Feldman HH, Jacova C, DeKosky ST, Barberger-Gateau P, Cummings J, et al Research criteria for the diagnosis of Alzheimer’s disease: revising the NINCDS–ADRDA criteria. The Lancet Neurology. 2007;6: 734–746. 10.1016/S1474-4422(07)70178-3 17616482

[6] Albert MS, DeKosky ST, Dickson D, Dubois B, Feldman HH, Fox NC, et al The diagnosis of mild cognitive impairment due to Alzheimer’s disease: Recommendations from the National Institute on Aging-Alzheimer’s Association workgroups on diagnostic guidelines for Alzheimer’s disease. Alzheimer’s & Dementia. 2011;7: 270–279. 10.1016/j.jalz.2011.03.008 21514249 PMC3312027

[7] Petersen RC, Caracciolo B, Brayne C, Gauthier S, Jelic V, Fratiglioni L. Mild cognitive impairment: a concept in evolution. Journal of Internal Medicine. 2014;275: 214–228. 10.1111/joim.12190 24605806 PMC3967548

[8] Mitchell AJ, Shiri-Feshki M. Rate of progression of mild cognitive impairment to dementia—meta-analysis of 41 robust inception cohort studies. Acta Psychiatrica Scandinavica. 2009;119: 252–265. 10.1111/j.1600-0447.2008.01326.x 19236314

[9] Petersen RC, Roberts RO, Knopman DS, Boeve BF, Geda YE, Ivnik RJ, et al Mild Cognitive Impairment: Ten Years Later. Archives of Neurology. 2009;66 10.1001/archneurol.2009.266 20008648 PMC3081688

[10] Artero S, Petersen R, Touchon J, Ritchie K. Revised Criteria for Mild Cognitive Impairment: Validation within a Longitudinal Population Study. Dementia and Geriatric Cognitive Disorders. 2006;22: 465–470. 10.1159/000096287 17047325

[11] Petersen RC. Mild cognitive impairment as a diagnostic entity. Journal of Internal Medicine. 2004;256: 183–194. 10.1111/j.1365-2796.2004.01388.x 15324362

[12] Wee C-Y, Yap P-T, Shen D, for the Alzheimer’s Disease Neuroimaging Initiative. Prediction of Alzheimer’s disease and mild cognitive impairment using cortical morphological patterns: Prediction of AD and MCI using Cortical Morphological Patterns. Human Brain Mapping. 2013;34: 3411–3425. 10.1002/hbm.22156 22927119 PMC3511623

[13] Cuingnet R, Gerardin E, Tessieras J, Auzias G, Lehéricy S, Habert M-O, et al Automatic classification of patients with Alzheimer’s disease from structural MRI: A comparison of ten methods using the ADNI database. NeuroImage. 2011;56: 766–781. 10.1016/j.neuroimage.2010.06.013 20542124

[14] Querbes O, Aubry F, Pariente J, Lotterie J-A, Démonet J-F, Duret V, et al Early diagnosis of Alzheimer’s disease using cortical thickness: impact of cognitive reserve. Brain. 2009;132: 2036–2047. 10.1093/brain/awp105 19439419 PMC2714060

[15] Beheshti I, Demirel H. Feature-ranking-based Alzheimer’s disease classification from structural MRI. Magnetic Resonance Imaging. 2016;34: 252–263. 10.1016/j.mri.2015.11.009 26657976

[16] Beheshti I, Demirel H. Probability distribution function-based classification of structural MRI for the detection of Alzheimer’s disease. Computers in Biology and Medicine. 2015;64: 208–216. 10.1016/j.compbiomed.2015.07.006 26226415

[17] Voevodskaya O, Simmons A, Nordenskjold R, Kullberg J, Ahlstrom H, Lind L, et al The effects of intracranial volume adjustment approaches on multiple regional MRI volumes in healthy aging and Alzheimer’s disease. Frontiers in Aging Neuroscience. 2014;6 10.3389/fnagi.2014.00006 25339897 PMC4188138

[18] Nozadi SH, Kadoury S, The Alzheimer’s Disease Neuroimaging Initiative. Classification of Alzheimer’s and MCI Patients from Semantically Parcelled PET Images: A Comparison between AV45 and FDG-PET. International Journal of Biomedical Imaging. 2018;2018: 1–13. 10.1155/2018/1247430 29736165 PMC5875062

[19] Li Y, Rinne JO, Mosconi L, Pirraglia E, Rusinek H, DeSanti S, et al Regional analysis of FDG and PIB-PET images in normal aging, mild cognitive impairment, and Alzheimer’s disease. European Journal of Nuclear Medicine and Molecular Imaging. 2008;35: 2169–2181. 10.1007/s00259-008-0833-y 18566819 PMC2693402

[20] Shaw LM, Vanderstichele H, Knapik-Czajka M, Clark CM, Aisen PS, Petersen RC, et al Cerebrospinal fluid biomarker signature in Alzheimer’s disease neuroimaging initiative subjects. Annals of Neurology. 2009;65: 403–413. 10.1002/ana.21610 19296504 PMC2696350

[21] Herukka S-K, Simonsen AH, Andreasen N, Baldeiras I, Bjerke M, Blennow K, et al Recommendations for cerebrospinal fluid Alzheimer’s disease biomarkers in the diagnostic evaluation of mild cognitive impairment. Alzheimer’s & Dementia. 2017;13: 285–295. 10.1016/j.jalz.2016.09.009 28341066

[22] Zhang D, Shen D, Alzheimer’s Disease Neuroimaging Initiative. Predicting Future Clinical Changes of MCI Patients Using Longitudinal and Multimodal Biomarkers. Chen K, editor. PLoS ONE. 2012;7: e33182 10.1371/journal.pone.0033182 22457741 PMC3310854

[23] Zhang D, Wang Y, Zhou L, Yuan H, Shen D. Multimodal classification of Alzheimer’s disease and mild cognitive impairment. NeuroImage. 2011;55: 856–867. 10.1016/j.neuroimage.2011.01.008 21236349 PMC3057360

[24] Lu D, Popuri K, Ding GW, Balachandar R, Beg MF. Multimodal and Multiscale Deep Neural Networks for the Early Diagnosis of Alzheimer’s Disease using structural MR and FDG-PET images. Scientific Reports. 2018;8 10.1038/s41598-017-18329-3 29632364 PMC5890270

[25] Forlenza OV, Radanovic M, Talib LL, Aprahamian I, Diniz BS, Zetterberg H, et al Cerebrospinal fluid biomarkers in Alzheimer’s disease: Diagnostic accuracy and prediction of dementia. Alzheimer’s & Dementia: Diagnosis, Assessment & Disease Monitoring. 2015;1: 455–463. 10.1016/j.dadm.2015.09.003 27239524 PMC4879480

[26] Young J, Modat M, Cardoso MJ, Mendelson A, Cash D, Ourselin S. Accurate multimodal probabilistic prediction of conversion to Alzheimer’s disease in patients with mild cognitive impairment. NeuroImage: Clinical. 2013;2: 735–745. 10.1016/j.nicl.2013.05.004 24179825 PMC3777690

[27] Westman E, Muehlboeck J-S, Simmons A. Combining MRI and CSF measures for classification of Alzheimer’s disease and prediction of mild cognitive impairment conversion. NeuroImage. 2012;62: 229–238. 10.1016/j.neuroimage.2012.04.056 22580170

[28] Weiner MW, Veitch DP, Aisen PS, Beckett LA, Cairns NJ, Cedarbaum J, et al 2014 Update of the Alzheimer’s Disease Neuroimaging Initiative: A review of papers published since its inception. Alzheimer’s & Dementia. 2015;11: e1–e120. 10.1016/j.jalz.2014.11.001 26073027 PMC5469297

[29] Wolz R, Julkunen V, Koikkalainen J, Niskanen E, Zhang DP, Rueckert D, et al Multi-Method Analysis of MRI Images in Early Diagnostics of Alzheimer’s Disease. Oreja-Guevara C, editor. PLoS ONE. 2011;6: e25446 10.1371/journal.pone.0025446 22022397 PMC3192759

[30] Sørensen L, Igel C, Pai A, Balas I, Anker C, Lillholm M, et al Differential diagnosis of mild cognitive impairment and Alzheimer’s disease using structural MRI cortical thickness, hippocampal shape, hippocampal texture, and volumetry. NeuroImage: Clinical. 2017;13: 470–482. 10.1016/j.nicl.2016.11.025 28119818 PMC5237821

[31] Gupta Y, Lee KH, Choi KY, Lee JJ, Kim BC, Kwon G-R. Alzheimer’s Disease Diagnosis Based on Cortical and Subcortical Features. Journal of Healthcare Engineering. 2019;2019: 1–13. 10.1155/2019/2492719 30944718 PMC6421724

[32] Lama RK, Gwak J, Park J-S, Lee S-W. Diagnosis of Alzheimer’s Disease Based on Structural MRI Images Using a Regularized Extreme Learning Machine and PCA Features. Journal of Healthcare Engineering. 2017;2017: 1–11. 10.1155/2017/5485080 29065619 PMC5494120

[33] Lebedeva AK, Westman E, Borza T, Beyer MK, Engedal K, Aarsland D, et al MRI-Based Classification Models in Prediction of Mild Cognitive Impairment and Dementia in Late-Life Depression. Frontiers in Aging Neuroscience. 2017;9 10.3389/fnagi.2017.00009 28210220 PMC5288688

[34] Wang W-Y, Yu J-T, Liu Y, Yin R-H, Wang H-F, Wang J, et al Voxel-based meta-analysis of grey matter changes in Alzheimer’s disease. Translational Neurodegeneration. 2015;4 10.1186/2047-9158-4-4 25834730 PMC4381413

[35] Matsuda H. Voxel-based Morphometry of Brain MRI in Normal Aging and Alzheimer’s Disease. Aging and Disease. 2013;4: 29–37. 23423504 PMC3570139

[36] Guo X, Wang Z, Li K, Li Z, Qi Z, Jin Z, et al Voxel-based assessment of gray and white matter volumes in Alzheimer’s disease. Neuroscience Letters. 2010;468: 146–150. 10.1016/j.neulet.2009.10.086 19879920 PMC2844895

[37] Hwang J, Kim CM, Jeon S, Lee JM, Hong YJ, Roh JH, et al Prediction of Alzheimer’s disease pathophysiology based on cortical thickness patterns. Alzheimer’s & Dementia: Diagnosis, Assessment & Disease Monitoring. 2016;2: 58–67. 10.1016/j.dadm.2015.11.008 27239533 PMC4879518

[38] Desikan RS, Cabral HJ, Hess CP, Dillon WP, Glastonbury CM, Weiner MW, et al Automated MRI measures identify individuals with mild cognitive impairment and Alzheimer’s disease. Brain. 2009;132: 2048–2057. 10.1093/brain/awp123 19460794 PMC2714061

[39] Zheng W, Yao Z, Hu B, Gao X, Cai H, et al Novel Cortical Thickness Pattern for Accurate Detection of Alzheimer’s Disease. Zhang Z, editor. Journal of Alzheimer’s Disease. 2015;48: 995–1008. 10.3233/JAD-150311 26444768

[40] Chupin M, Gérardin E, Cuingnet R, Boutet C, Lemieux L, Lehéricy S, et al Fully automatic hippocampus segmentation and classification in Alzheimer’s disease and mild cognitive impairment applied on data from ADNI. Hippocampus. 2009;19: 579–587. 10.1002/hipo.20626 19437497 PMC2837195

[41] Boutet C, Chupin M, Lehéricy S, Marrakchi-Kacem L, Epelbaum S, Poupon C, et al Detection of volume loss in hippocampal layers in Alzheimer’s disease using 7 T MRI: A feasibility study. NeuroImage: Clinical. 2014;5: 341–348. 10.1016/j.nicl.2014.07.011 25161900 PMC4141975

[42] Ben Ahmed O, Benois-Pineau J, Allard M, Ben Amar C, Catheline G. Classification of Alzheimer’s disease subjects from MRI using hippocampal visual features. Multimedia Tools and Applications. 2015;74: 1249–1266. 10.1007/s11042-014-2123-y

[43] Sørensen L, Igel C, Liv Hansen N, Osler M, Lauritzen M, Rostrup E, et al Early detection of Alzheimer’s disease using MRI hippocampal texture: Early AD Detection Using Hippocampal Texture. Human Brain Mapping. 2016;37: 1148–1161. 10.1002/hbm.23091 26686837 PMC6867374

[44] Korolev IO, Symonds LL, Bozoki AC, Alzheimer’s Disease Neuroimaging Initiative. Predicting Progression from Mild Cognitive Impairment to Alzheimer’s Dementia Using Clinical, MRI, and Plasma Biomarkers via Probabilistic Pattern Classification. Herholz K, editor. PLOS ONE. 2016;11: e0138866 10.1371/journal.pone.0138866 26901338 PMC4762666

[45] Kälin AM, Park MTM, Chakravarty MM, Lerch JP, Michels L, Schroeder C, et al Subcortical Shape Changes, Hippocampal Atrophy and Cortical Thinning in Future Alzheimer’s Disease Patients. Frontiers in Aging Neuroscience. 2017;9 10.3389/fnagi.2017.00009 28326033 PMC5339600

[46] Long X, Chen L, Jiang C, Zhang L, Alzheimer’s Disease Neuroimaging Initiative. Prediction and classification of Alzheimer disease based on quantification of MRI deformation. Chen K, editor. PLOS ONE. 2017;12: e0173372 10.1371/journal.pone.0173372 28264071 PMC5338815

[47] Morris JC. The Clinical Dementia Rating (CDR): Current version and scoring rules. Neurology. 1993;43: 2412–2412. 10.1212/WNL.43.11.2412-a 8232972

[48] Seo EH, Kim H, Lee KH, Choo IH. Altered Executive Function in Pre-Mild Cognitive Impairment. Seo SW, editor. Journal of Alzheimer’s Disease. 2016;54: 933–940. 10.3233/JAD-160052 27567814

[49] Seo EH, Kim H, Choi KY, Lee KH, Choo IH. Association of subjective memory complaint and depressive symptoms with objective cognitive functions in prodromal Alzheimer’s disease including pre-mild cognitive impairment. Journal of Affective Disorders. 2017;217: 24–28. 10.1016/j.jad.2017.03.062 28380342

[50] Elwood RW. The Wechsler Memory Scale Revised: Psychometric characteristics and clinical application. Neuropsychology Review. 1991;2: 179–201. 10.1007/BF01109053 1844708

[51] McKhann G, Drachman D, Folstein M, Katzman R, Price D, Stadlan EM. Clinical diagnosis of Alzheimer’s disease: report of the NINCDS-ADRDA Work Group under the auspices of Department of Health and Human Services Task Force on Alzheimer's Disease. Neurology. 1984 7; 34: 939–944. 10.1212/wnl.34.7.939 6610841

[52] Tustison NJ, Avants BB, Cook PA, Yuanjie Zheng, Egan A, Yushkevich PA, et al N4ITK: Improved N3 Bias Correction. IEEE Transactions on Medical Imaging. 2010;29: 1310–1320. 10.1109/TMI.2010.2046908 20378467 PMC3071855

[53] Ashburner J, Friston KJ. Why Voxel-Based Morphometry Should Be Used. NeuroImage. 2001;14: 1238–1243. 10.1006/nimg.2001.0961 11707080

[54] Whitwell JL. Voxel-Based Morphometry: An Automated Technique for Assessing Structural Changes in the Brain. Journal of Neuroscience. 2009;29: 9661–9664. 10.1523/JNEUROSCI.2160-09.2009 19657018 PMC6666603

[55] Schmitter D, Roche A, Maréchal B, Ribes D, Abdulkadir A, Bach-Cuadra M, et al An evaluation of volume-based morphometry for prediction of mild cognitive impairment and Alzheimer’s disease. NeuroImage: Clinical. 2015;7: 7–17. 10.1016/j.nicl.2014.11.001 25429357 PMC4238047

[56] Xiao Z, Ding Y, Lan T, Zhang C, Luo C, Qin Z. Brain MR Image Classification for Alzheimer’s Disease Diagnosis Based on Multifeature Fusion. Computational and Mathematical Methods in Medicine. 2017;2017: 1–13. 10.1155/2017/1952373 28611848 PMC5458434

[57] Maintz JBA, Viergever MA. A survey of medical image registration. Med Image Anal. 1998; 2: 1–36. 10.1016/S1361-8415(01)80026-8 10638851

[58] Fischl B, Salat DH, Busa E, Albert M, Dieterich M, Haselgrove C, et al Whole Brain Segmentation. Neuron. 2002;33: 341–355. 10.1016/s0896-6273(02)00569-x 11832223

[59] Fischl B. FreeSurfer. NeuroImage. 2012;62: 774–781. 10.1016/j.neuroimage.2012.01.021 22248573 PMC3685476

[60] Dale AM, Fischl B, Sereno MI. Cortical Surface-Based Analysis. Neuroimage. 1999; 9: 179–194. 10.1006/nimg.1998.0395 9931268

[61] Iglesias JE, Augustinack JC, Nguyen K, Player CM, Player A, Wright M, et al A computational atlas of the hippocampal formation using ex vivo, ultra-high resolution MRI: Application to adaptive segmentation of in vivo MRI. NeuroImage. 2015;115: 117–137. 10.1016/j.neuroimage.2015.04.042 25936807 PMC4461537

[62] Saygin ZM, Kliemann D, Iglesias JE, van der Kouwe AJW, Boyd E, Reuter M, et al High-resolution magnetic resonance imaging reveals nuclei of the human amygdala: manual segmentation to automatic atlas. NeuroImage. 2017;155: 370–382. 10.1016/j.neuroimage.2017.04.046 28479476 PMC5557007

[63] Jack CR, Barkhof F, Bernstein MA, Cantillon M, Cole PE, DeCarli C, et al Steps to standardization and validation of hippocampal volumetry as a biomarker in clinical trials and diagnostic criterion for Alzheimer’s disease. Alzheimer’s & Dementia. 2011;7: 474–485.e4. 10.1016/j.jalz.2011.04.007 21784356 PMC3396131

[64] Hunsaker MR, Rosenberg JS, Kesner RP. The role of the dentate gyrus, CA3a,b, and CA3c for detecting spatial and environmental novelty. Hippocampus. 2008;18: 1064–1073. 10.1002/hipo.20464 18651615

[65] Andersen AH, Gash DM, Avison MJ. Principal component analysis of the dynamic response measured by fMRI: a generalized linear systems framework. Magnetic Resonance Imaging. 1999;17: 795–815. 10.1016/s0730-725x(99)00028-4 10402587

[66] Lebedev AV, Westman E, Van Westen GJP, Kramberger MG, Lundervold A, Aarsland D, et al Random Forest ensembles for detection and prediction of Alzheimer’s disease with a good between-cohort robustness. NeuroImage: Clinical. 2014;6: 115–125. 10.1016/j.nicl.2014.08.023 25379423 PMC4215532

[67] Chen X, Ishwaran H. Random forests for genomic data analysis. Genomics. 2012;99: 323–329. 10.1016/j.ygeno.2012.04.003 22546560 PMC3387489

[68] Zhang S, Li X, Zong M, Zhu X, Wang R. Efficient kNN Classification with Different Numbers of Nearest Neighbors. IEEE Transactions on Neural Networks and Learning Systems. 2018;29: 1774–1785. 10.1109/TNNLS.2017.2673241 28422666

[69] Mohammed A, Al-Azzo F, Milanova M. Classification of Alzheimer Disease based on Normalized Hu Moment Invariants and Multiclassifier. International Journal of Advanced Computer Science and Applications. 2017;8 10.14569/IJACSA.2017.081102

[70] Cohen J. A Coefficient of Agreement for Nominal Scales. Educational and Psychological Measurement. 1960;20: 37–46. 10.1177/001316446002000104

[71] Jha D, Alam S, Pyun J-Y, Lee KH, Kwon G-R. Alzheimer’s Disease Detection Using Extreme Learning Machine, Complex Dual Tree Wavelet Principal Coefficients and Linear Discriminant Analysis. Journal of Medical Imaging and Health Informatics. 2018;8: 881–890. 10.1166/jmihi.2018.2381

[72] Beheshti I, Demirel H, Farokhian F, Yang C, Matsuda H. Structural MRI-based detection of Alzheimer’s disease using feature ranking and classification error. Computer Methods and Programs in Biomedicine. 2016;137: 177–193. 10.1016/j.cmpb.2016.09.019 28110723

